# Blunted Cortisol Awakening Response in Patients with Inflammatory Bowel Disease: A Cross-Sectional Case–Control Study

**DOI:** 10.3390/medsci14040463

**Published:** 2026-08-07

**Authors:** Marija Rafaela Plosnic Todoric, Roko Santic, Marko Kumric, Piero Marin Zivkovic, Ivna Olic, Doris Rusic, Marino Vilovic, Ivan Males, Daniela Supe Domic, Josko Bozic

**Affiliations:** 1 Department of Neonatology, University Hospital of Split, Spinciceva 1, 21000 Split, Croatia; rafaela.plosnic@gmail.com; 2 Evidence Based Medicine Doctoral Study, University of Split School of Medicine, Soltanska 2A, 21000 Split, Croatia; 3 Department of Pathophysiology, University of Split School of Medicine, Soltanska 2A, 21000 Split, Croatia; roko.santic@mefst.hr (R.S.); marko.kumric@mefst.hr (M.K.); marino.vilovic@mefst.hr (M.V.); 4 Laboratory for Cardiometabolic Research, University of Split School of Medicine, Soltanska 2A, 21000 Split, Croatia; 5 Department of Gastroenterology and Hepatology, University Hospital of Split, Spinciceva 1, 21000 Split, Croatia; pzivkovic@kbsplit.hr (P.M.Z.); ivna1211@gmail.com (I.O.); 6 Department of Pharmacy, University of Split School of Medicine, Soltanska 2A, 21000 Split, Croatia; doris.rusic@mefst.hr; 7 Department of Abdominal Surgery, University Hospital of Split, Spinciceva 1, 21000 Split, Croatia; ivmales@kbsplit.hr; 8 Faculty of Health Studies, University of Split, Rudera Boskovica 35, 21000 Split, Croatia; 9 Department of Laboratory Diagnostics, University Hospital of Split, Spinciceva 1, 21000 Split, Croatia

**Keywords:** inflammatory bowel disease, cortisol awakening response, hypothalamic–pituitary–adrenal axis, salivary cortisol, diurnal cortisol slope, gut–brain axis, systemic inflammation, high-sensitivity C-reactive protein

## Abstract

Background: Inflammatory bowel disease (IBD) involves chronic immune activation, yet alterations in the cortisol awakening response (CAR) remain poorly defined. We compared cortisol dynamics in patients with IBD and healthy controls and explored associations with phenotype, endoscopic activity, and inflammation. Methods: This single-centre, cross-sectional case–control study enrolled 100 patients with IBD (51 with ulcerative colitis [UC] and 49 with Crohn’s disease [CD]) and 78 frequency-matched controls. Salivary cortisol was measured at awakening (C0), 30–45 min later (C1), and bedtime (C2); 97 patients and 78 controls met timing criteria. The absolute post-awakening increment (ΔCAR) and area under the curve with respect to increase (AUCi) were calculated. Analyses included non-parametric tests, adjusted regression, mixed models, and multiplicity-corrected within-IBD comparisons and correlations. Results: Patients with IBD had lower C0 (median 0.478 vs. 1.067 µg/dL) and C1 (0.850 vs. 1.950 µg/dL; both *p* < 0.001), whereas C2 was comparable (*p* = 0.819). ΔCAR (0.130 vs. 0.461 µg/dL) and AUCi (2.080 vs. 7.770 min·µg/dL) were lower in IBD (both *p* < 0.001); adjusted analyses confirmed this pattern. Cortisol metrics did not differ consistently by IBD subtype or endoscopic activity. Higher high-sensitivity C-reactive protein (hs-CRP) correlated positively with C0 (ρ = 0.325; q = 0.041) and inversely with ΔCAR (ρ = −0.402; q = 0.002), AUCi (ρ = −0.408; q = 0.002), and relative CAR (ρ = −0.517; q < 0.001). Conclusions: IBD was associated with attenuated CAR and lower morning cortisol output, with preserved bedtime concentrations. These cross-sectional findings support a relationship between systemic inflammation and altered early-morning HPA-axis dynamics but do not establish causality.

## 1. Introduction

Inflammatory bowel disease (IBD), principally Crohn’s disease (CD) and ulcerative colitis (UC), comprises chronic, relapsing immune-mediated disorders of the gastrointestinal tract [1,2]. Current pathophysiological models conceptualise IBD as a systems-level disorder arising from interactions among host genetic susceptibility, epithelial barrier dysfunction, intestinal microbial perturbation, environmental exposures, and dysregulated innate and adaptive immune responses [3,4,5,6]. In parallel, therapeutic goals have evolved from symptom control alone toward treat-to-target strategies that include clinical remission, normalisation of inflammatory biomarkers, endoscopic healing, preservation of quality of life (QoL), and reduction of disability [7,8].

Within this broader framework, the brain–gut axis has become increasingly relevant to IBD pathophysiology and clinical outcomes. Bidirectional communication among the central nervous system, autonomic and enteric nervous systems, hypothalamic–pituitary–adrenal (HPA) axis, intestinal microbiota, and mucosal immune pathways provides a plausible biological route by which psychological state and intestinal inflammation may influence one another [9,10,11,12]. Clinically, symptoms of anxiety and depression are common in IBD, with meta-analytic estimates suggesting anxiety symptoms in approximately one-third and depressive symptoms in approximately one-quarter of patients [13]. Systematic-review evidence further supports psychological stress as a potential contributor to disease course and poor psychosocial outcomes, although the relative contributions of inflammatory, microbial, behavioural, treatment-related, and psychological mechanisms remain incompletely resolved [14]. Longitudinal evidence also supports bidirectional effects: intestinal inflammatory activity may worsen subsequent psychological health, while anxiety and depression may adversely affect IBD prognosis [15].

The HPA axis is a central neuroendocrine regulator of the stress–immune interface. In response to psychological and inflammatory stressors, hypothalamic corticotropin-releasing hormones and pituitary adrenocorticotropic hormones stimulate adrenal glucocorticoid secretion. Cortisol, the principal human glucocorticoid, acts through glucocorticoid receptors expressed across immune and non-immune tissues and contributes to inflammatory regulation by modulating cytokine production, leukocyte trafficking, epithelial barrier biology, and negative feedback control of the stress response [16,17,18]. However, chronic stress and persistent inflammatory signalling may reduce glucocorticoid receptor sensitivity or uncouple cortisol availability from inflammatory demand, producing a state of relative glucocorticoid resistance despite ongoing immune activation [19]. Recent experimental work has strengthened the biological plausibility of stress–gut inflammatory coupling by showing that stress-related glucocorticoid signalling can alter enteric glial, neuronal, myeloid-cell, and epithelial-stem-cell pathways and thereby aggravate colitis-like inflammation [20,21].

The cortisol awakening response (CAR) is conventionally defined as the rise in free cortisol concentrations during the first 30–45 min after morning awakening and is commonly assessed using self-collected saliva samples [22,23]. In contrast to a single morning cortisol value, CAR-based assessment separates awakening cortisol from the post-awakening increase and can therefore capture complementary aspects of early-morning HPA-axis activity. Nevertheless, CAR should be interpreted as a timing-sensitive marker of post-awakening cortisol dynamics rather than as a simple proxy for acute psychological stress reactivity. Expert consensus and subsequent methodological updates emphasise strict sampling timing, participant instruction, covariate control, and preferably objective adherence verification, because delayed or mistimed samples can substantially bias CAR estimates [24,25]. Standard CAR quantification includes the absolute post-awakening increment, the area under the curve with respect to increase (AUCi), which indexes time-dependent change, and the area under the curve with respect to ground (AUCg), which indexes total cortisol exposure over the sampled interval [26]. Recent high-resolution interstitial cortisol work has also raised caution about whether the post-awakening rise reflects awakening-induced secretion per se or the continuation of an underlying circadian rise, further underscoring the need for careful operational definition and conservative interpretation [27].

Despite strong mechanistic reasons to expect HPA-axis involvement in IBD, CAR-specific evidence remains limited and methodologically heterogeneous. Earlier neuroendocrine work suggested that the sympathetic nervous system and HPA axis, which appear coupled in healthy controls, may be uncoupled in patients with IBD [28]. More recently, a prospective study of patients with CD in clinical remission reported a blunted CAR compared with healthy controls, in the context of cognitive and inflammatory alterations [29]. A separate pilot study found that morning salivary cortisol was associated with anxiety symptom severity in UC but did not identify clear associations with CRP, faecal calprotectin, or endoscopic activity [30]. Thus, existing data support the plausibility of altered HPA-axis function in IBD but do not establish whether IBD is characterised primarily by lower awakening cortisol, an attenuated post-awakening rise, reduced early-morning cortisol output, or broader changes in waking-day cortisol exposure. It also remains unclear whether such alterations differ between UC and CD, track endoscopic disease activity, or correlate with objective inflammatory biomarkers.

## 2. Materials and Methods

### 2.1. Study Design and Ethics

This was a single-centre, cross-sectional, frequency-matched case–control study conducted at the Department of Gastroenterology, University Hospital of Split (KBC Split), Split, Croatia. Patient recruitment was carried out between April 2025 and April 2026. The study protocol was approved by the Ethics Committee of KBC Split (class: 520-03/25-01/102; number: 2181-147/01-06/LJ.Z.-25-02; date of approval: 31 March 2025). All participants provided written informed consent before enrolment. Eligible patients were screened consecutively in the outpatient gastroenterology clinic to reduce clinician-driven selection by IBD subtype, disease activity, treatment status, or expected cortisol profile.

The primary aim of this cross-sectional case–control study was to compare salivary cortisol at awakening, 30–45 min post-awakening, and bedtime between patients with IBD and healthy controls and to derive complementary CAR metrics using recorded sampling intervals. We hypothesised that patients with IBD would exhibit a blunted CAR compared with healthy controls, consistent with attenuated early-morning HPA-axis dynamics. Secondary objectives were to explore whether cortisol metrics differed between UC and CD, between score-defined active disease and remission, and whether inflammatory biomarkers—including C-reactive protein, albumin, and faecal calprotectin—were associated with CAR-derived outcomes.

### 2.2. Participants, Controls, Matching, and Screening

Adult patients were eligible if they had established inflammatory bowel disease (IBD), comprising UC or CD, with disease duration of at least one year before enrolment. IBD diagnosis followed ECCO and ECCO-ESGAR guidance and was based on clinical, biochemical, endoscopic, histological, and radiological data when indicated [31,32,33,34].

Controls had no gastrointestinal, inflammatory, autoimmune, or endocrine disease, were screened using the same applicable exclusion criteria, and were frequency-matched to IBD patients by age, sex, and BMI. Because matching was not individual, analyses used independent-group comparisons.

Exclusion criteria for both groups were age < 18 or >75 years; systemic corticosteroid use within the preceding three months; current use of inhaled corticosteroids, intranasal corticosteroids, rectal corticosteroids, budesonide, or oral contraceptives; confirmed adrenal insufficiency; pregnancy or lactation; active malignancy; acute active infection at the time of sampling; inability to provide informed consent; or inability to comply with the saliva-sampling protocol. Medication-related exclusion criteria were assessed by participant interview and review of available medical documentation. Accordingly, no enrolled participant had used systemic corticosteroids within the preceding three months or was receiving inhaled, intranasal, rectal, or budesonide therapy at the time of cortisol assessment; these prespecified exclusions were intended to minimise direct pharmacological confounding of HPA-axis measurements.

Depressive symptoms were assessed with the PHQ-9 to characterise potential HPA-axis confounding, but PHQ-9 scores were not included in the prespecified cortisol models or analysed as study outcomes [35]. The PHQ-9 was not treated as a measure of sleep quality, and no validated sleep questionnaire was administered.

The enrolled cohort comprised 178 participants: 100 IBD patients, including 51 UC and 49 CD patients, and 78 healthy controls. The full enrolled cohort was used for non-time-dependent descriptive, clinical, biochemical, and serum cortisol analyses. CAR-derived and other time-dependent salivary cortisol analyses used the timing-valid analytical sample defined below.

### 2.3. Clinical, Anthropometric, and Endoscopic Assessment

At enrolment, all participants underwent a standardised clinical assessment. Relevant items from current and past medical history were obtained during the screening visit, including comorbidities, current medication use, recent infection, corticosteroid exposure, smoking status, and factors potentially affecting salivary cortisol sampling. Use of selective serotonin reuptake inhibitors and serotonin–noradrenaline reuptake inhibitors was specifically verified from the available medication histories; no enrolled participant was receiving either medication class at the time of cortisol assessment. In patients with IBD, disease subtype was classified as UC or CD, disease duration was recorded, current IBD treatment was documented, and previous IBD-related surgery was recorded.

Height, body mass, waist circumference, and hip circumference were measured by trained study personnel using standardised procedures. BMI and waist-to-hip ratio were calculated from these measurements.

Disease activity was defined using subtype-specific endoscopic criteria rather than clinician global assessment alone. Colonoscopy or ileocolonoscopy was performed by an experienced gastroenterologist at enrolment or within two weeks of saliva sampling. Endoscopic scoring was performed before cortisol-derived outcomes were analysed.

To reduce endoscopic misclassification, scoring was reviewed within a quality-control process based on standard agreement frameworks, and disagreements affecting activity classification were resolved by consensus [36,37].

For UC, endoscopic activity was assessed using the Mayo endoscopic subscore (MES) [38,39] and the Ulcerative Colitis Endoscopic Index of Severity (UCEIS) [40,41]. Score-defined active UC was defined as MES ≥ 2; patients with MES < 2 were classified as being in score-defined remission. For CD, endoscopic activity was assessed using the Simple Endoscopic Score for Crohn’s Disease (SES-CD) [42]. Score-defined active CD was defined as SES-CD ≥ 3; patients with SES-CD < 3 were classified as being in score-defined remission. CDAI and Harvey–Bradshaw Index values were recorded descriptively where available [43,44] but were not used to define CD activity in the present analyses. UC and CD endoscopic indices were not pooled as continuous measures because the instruments are not interchangeable across disease subtypes.

### 2.4. Fasting Blood Sampling, Biochemical Analyses, and Faecal Calprotectin

Fasting venous blood was collected the morning after saliva sampling and used for biochemical markers, hs-CRP, and serum cortisol.

Blood analyses included complete blood count, renal and hepatic biochemistry, glucose, iron status, albumin, lipid parameters, high-sensitivity C-reactive protein (hs-CRP), serum cortisol, adrenocorticotropic hormone (ACTH), and corticosteroid-binding globulin (CBG). Plasma hs-CRP concentrations were measured by a latex-enhanced turbidimetric method using Abbott Laboratories reagents (Chicago, IL, USA). Other biochemical analyses were performed using standard validated procedures in the institutional clinical laboratory.

Morning serum cortisol was measured from the same fasting next-morning venous blood sample using electrochemiluminescence immunoassay (ECLIA) on the Cobas e801 platform (Roche Diagnostics GmbH, Mannheim, Germany). Adrenocorticotropic hormone (ACTH) and corticosteroid-binding globulin (CBG) were additionally measured during the same fasting next-morning blood sampling episode using validated institutional laboratory procedures. ACTH and CBG concentrations are reported in pg/mL and mg/L, respectively. Serum cortisol values are reported in nmol/L. Because serum cortisol was obtained on the morning after salivary sampling, it was analysed as a complementary fasting morning serum measure rather than as a time-matched equivalent of salivary C0 or C1.

Faecal calprotectin was measured in all patients with IBD and in 10 controls selected at random before cortisol and other laboratory results were available. Because only a small control subset had faecal calprotectin data, between-group inference for this marker was not performed, and biomarker–cortisol correlations were restricted to the IBD cohort.

### 2.5. IBDQ-32 Assessment

Disease-related QoL was assessed in IBD patients using the Croatian-language form of the 32-item Inflammatory Bowel Disease Questionnaire (IBDQ-32) [45]. The questionnaire was completed during the same study assessment episode as clinical phenotyping and biochemical assessment. IBDQ-32 total scores range from 32 to 224, with higher scores indicating better disease-related QoL. Exploratory analyses used complete available total-score records, without imputation [45].

### 2.6. Participant Instruction and Saliva-Collection Materials

Participants received face-to-face and written instructions emphasising exact sampling times and completed a standardised sampling-time log.

Each participant received a sealed saliva-collection pack containing three individually labelled Salivette^®^ Cortisol collection devices with polypropylene collection tubes and synthetic swabs (Sarstedt, Nümbrecht, Germany), one for each sampling time point: awakening (C0), 30–45 min after awakening (C1), and bedtime (C2). The pack also contained the written sampling protocol, the time-recording sheet, and instructions for refrigerated storage and handover.

### 2.7. Salivary Cortisol Collection Protocol

Saliva samples were collected at three time points: immediately upon awakening (C0), 30–45 min after awakening (C1), and immediately before bedtime (C2). There was no additional post-awakening saliva sample; therefore, the protocol comprised exactly three salivary cortisol time points. Saliva was collected according to a non-stimulated saliva protocol using Salivette^®^ Cortisol devices with synthetic swabs.

The C0 sample was collected immediately after awakening, before food intake, tooth brushing, smoking, nicotine use, exercise, or prolonged physical activity. The C1 sample was collected 30–45 min after C0, again before eating, brushing teeth, smoking, nicotine use, or exercise whenever possible. The C2 sample was collected immediately before bedtime.

Participants rinsed their mouth with water before each saliva collection and waited briefly before placing the synthetic swab in the mouth. They were instructed not to collect saliva if visible oral bleeding was present. Any protocol deviations, unusual sleep patterns, acute illness symptoms, late sampling, uncertainty about timing, or other relevant events were to be recorded on the sampling log sheet. Participants were not specifically instructed to avoid shift work or atypical sleep schedules on the sampling day.

After each collection, samples were placed back in the labelled collection tubes and stored refrigerated at +2 °C to +8 °C until handover to the researcher. Participants returned all three samples and the completed sampling-time log at the next study visit, no later than two days after collection.

### 2.8. Laboratory Handling and Salivary Cortisol Assay

On receipt, samples were inspected, kept cold, and stored at −20 °C until analysis. Before assay, samples were equilibrated at room temperature for 60 min and centrifuged at 1500× *g* for 3 min to obtain clarified saliva suitable for automated immunoassay analysis.

Salivary cortisol was measured by immunochemiluminometric assay (CLIA) on the IDS-iSYS Multi-Discipline Automated System (IDS Ltd., Boldon, UK). The functional sensitivity of the assay was 0.02 µg/dL and the analytical measuring range was 0.02–3.00 µg/dL. Salivary cortisol values are reported in µg/dL according to IDS-iSYS calibration.

All salivary cortisol samples were measured in duplicate, and the mean of duplicate measurements was used for statistical analysis. Duplicate measurements and laboratory quality-control records were reviewed before database lock; values passing laboratory quality control were retained regardless of distributional extremity. No sample underwent more than one freeze–thaw cycle. All saliva samples were visually inspected for blood contamination before analysis; no sample showed visible blood contamination, and no sample was excluded for this reason. Laboratory personnel were blinded to group allocation and clinical phenotype.

### 2.9. Timing Quality Control and Cortisol-Derived Metrics

All recorded sampling times were converted to minutes since midnight. If the recorded bedtime sample time was numerically earlier than the awakening time on the 24 h clock, indicating sampling after midnight, 1440 min were added before elapsed intervals were calculated. The C0-to-C1 interval (dt1 = T1 − T0) and the C1-to-C2 interval (dt2 = T2 − T1) were computed from participant-recorded times and rounded to the nearest minute.

Timing logs were checked before database lock, and confirmed transcription errors were corrected. CAR analyses included only records with a C0-to-C1 interval of 30–45 min; three IBD records outside this window were excluded [24,25,26]. Thus, the time-dependent analytical sample comprised 97 IBD patients and 78 healthy controls. The full enrolled cohort remained available for non-time-dependent descriptive, clinical, biochemical, and serum cortisol analyses.

Because electronic time-stamp verification was not used, timing validity relied on participant logs supported by standardised instructions and prespecified exclusion rules.

CAR metrics were computed using actual recorded sampling intervals. The absolute cortisol awakening response was calculated as ΔCAR = C1 − C0 [22,23]. CAR-specific area under the curve with respect to increase (CAR-specific AUCi) was calculated as 0.5 × (C1 − C0) × dt1 over the C0-to-C1 window. CAR-specific area under the curve with respect to ground (CAR-specific AUCg) was calculated as 0.5 × (C0 + C1) × dt1 over the same window. Partial waking-to-bed AUCg was calculated as 0.5 × (C0 + C1) × dt1 + 0.5 × (C1 + C2) × dt2. AUC calculations followed the trapezoidal approach of Pruessner et al. [26].

Additional derived metrics included relative CAR = (C1 − C0)/C0 × 100%, C1/C2 ratio, and daily amplitude = C1 − C2. The post-awakening-to-bedtime diurnal cortisol slope (DCS) was calculated as [log_2_(C2) − log_2_(C1)] divided by the elapsed time in hours between C1 and C2. Less negative DCS values indicate a flatter post-awakening-to-bedtime cortisol decline. The non-standard sum C0 + C1 + C2 was not used because the three samples were not equally spaced.

### 2.10. Data Blinding, Database Lock, and Analytical Dataset Preparation

Clinical and laboratory data were entered into a de-identified study database. Before statistical analysis, sampling times, cortisol values, and clinical variables were checked for missingness, out-of-range values, transcription errors, and internal inconsistency. Corrections were made only by reference to original source documents, including laboratory reports and participant sampling logs, before database lock.

The statistical analyst received de-identified datasets. Group and clinical phenotype labels were masked during timing validation and cortisol-metric derivation and released only after exclusion rules and formulas were locked.

### 2.11. Sample Size

Assuming a moderate between-group effect size for CAR outcomes, d = 0.50, with α = 0.05 and 80% power, 64 participants per group were required. Allowing for approximately 20% non-analysable or missing data yielded a target of 77 participants per group.

The final enrolled cohort and the timing-valid analytical sample exceeded the minimum required sample size for the primary IBD-versus-control comparison. The study was not powered for formal equivalence testing or for subgroup analyses by IBD subtype, score-defined disease activity, or biological therapy status; these analyses were therefore considered exploratory.

**Figure 1:**
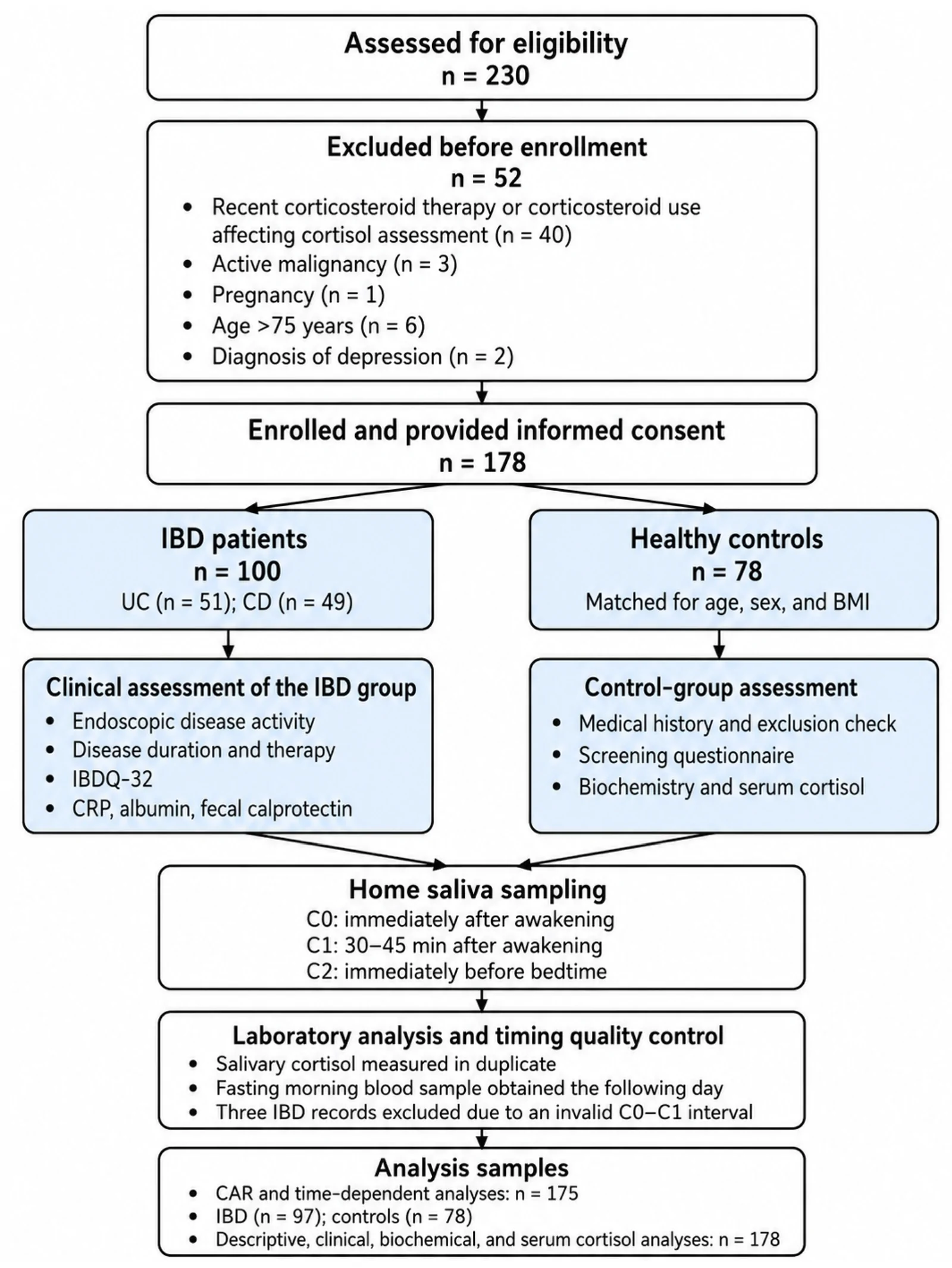
Study flow, assessment sequence, home saliva-sampling protocol, and final analytical samples. C0, awakening salivary cortisol; C1, salivary cortisol 30–45 min after awakening; C2, bedtime salivary cortisol; CAR, cortisol awakening response; CD, Crohn’s disease; IBD, inflammatory bowel disease; UC, ulcerative colitis.

The overall study flow, assessment sequence, home saliva-sampling protocol, and final analytical samples are summarised in Figure 1.

### 2.12. Statistical Analysis

Statistical analysis was performed using MedCalc Statistical Software, version 23.5.5 (MedCalc Software Ltd., Ostend, Belgium). Continuous variables were summarised as median [interquartile range, IQR] or mean ± standard deviation where appropriate and categorical variables as *n* (%). Normality was assessed visually and using the Shapiro–Wilk test. Between-group comparisons of non-normally distributed continuous variables were performed using the Mann–Whitney U test. Approximately normally distributed continuous variables were compared using Welch’s independent-samples *t*-test where appropriate. Categorical variables were compared using the chi-square test or Fisher’s exact test where expected cell counts were small. Analyses were performed using available cases for each variable; no imputation was performed for missing clinical, biochemical, endoscopic, cortisol, PHQ-9 screening, or IBDQ-32 data. Exact *p*-values were reported where appropriate; very small *p*-values were reported as *p* < 0.001.

Because salivary cortisol and AUC-derived outcomes were right-skewed, unadjusted comparisons used non-parametric tests. Additional multivariable sensitivity analyses were performed in Python 3.13.5 using statsmodels 0.14.6. Natural-log-transformed cortisol and AUC-derived outcomes were analysed using OLS regression with HC3 heteroscedasticity-consistent standard errors, adjusted for age, sex, BMI, wake time, and the C0-to-C1 sampling interval (dt1). Effect estimates from log-transformed models are reported as exponentiated IBD/control ratios.

The log(AUCi) model was restricted to positive AUCi records, whereas raw-scale HC3 and median quantile-regression models retained all available time-dependent records. A residualised post-awakening model additionally included log(C0) to assess whether group differences in log(C1) persisted after accounting for awakening cortisol. Median quantile regression (τ = 0.50) and a repeated-measures linear mixed model for log-transformed C0, C1, and C2, with participant-level random intercepts, were used as sensitivity analyses. The mixed model included IBD status, time point, the IBD-by-time-point interaction, age, sex, BMI, wake time, and dt1. DCS was analysed on its original log_2_-slope scale, with positive coefficients indicating a less negative, flatter post-awakening-to-bedtime decline. These additional models were treated as sensitivity analyses rather than independent primary outcome families. Endoscopic scoring quality control was evaluated using agreement statistics. Interobserver agreement between the primary and second gastroenterologist was assessed using weighted kappa for categorical classifications and intraclass correlation coefficients for continuous scores. Intraobserver agreement was assessed using the same approach after repeat scoring by the primary gastroenterologist.

Within-IBD subgroup comparisons were performed for disease subtype (UC vs. CD), endoscopic score-defined activity category (active disease vs. remission), and current biological therapy status. These analyses were exploratory. Biological therapy comparisons were performed among IBD patients with available treatment data and were not adjusted for treatment indication, disease severity, or prior treatment exposure.

Additional post hoc exploratory comparisons of the 20 demographic and biochemical variables presented in Table 1 were performed between patients with UC and CD in the full IBD cohort using the same variable-specific statistical tests described above. Benjamini–Hochberg false-discovery-rate correction was applied across the 20 comparisons, and both unadjusted *p*-values and adjusted q-values are reported in Supplementary Table S11.

Spearman rank correlations were used to examine associations between inflammatory biomarkers and cortisol parameters within the IBD cohort. hs-CRP was log_10_-transformed for correlation analyses. In accordance with the revised correction strategy, the global biomarker-cortisol correlation family comprised 14 biomarkers × 10 cortisol outcomes, yielding 140 tests in total. The 10 cortisol outcomes in this matrix were C0, C1, C2, ΔCAR, CAR-specific AUCi, CAR-specific AUCg, partial waking-to-bed AUCg, relative CAR, C1/C2 ratio, and serum cortisol. Daily amplitude was reported descriptively but was not included in this biomarker FDR matrix. Benjamini–Hochberg false-discovery-rate correction was applied globally across the 140 biomarker-cortisol tests. DCS was analysed as an additional exploratory time-dependent cortisol outcome and interpreted separately from the prespecified global biomarker–cortisol matrix unless explicitly stated.

Subtype-specific endoscopic-score correlations, including MES within UC and SES-CD within CD, were corrected within their respective exploratory disease-specific families. Exploratory IBDQ-32 analyses were performed within the IBD time-dependent analytical sample using complete available IBDQ-32 total-score records. Spearman correlations were calculated between IBDQ-32 total score and available cortisol outcomes, excluding DCS from the primary IBDQ-cortisol matrix unless separately specified. BH-FDR correction was applied across the IBDQ-cortisol tests. Statistical significance was defined as two-sided *p* < 0.05; FDR-adjusted q-values were interpreted as the primary basis for multiplicity-adjusted exploratory inference.

## 3. Results

### 3.1. Demographic and Biochemical Characteristics

Groups were well matched for age (39 [28–47] vs. 37 [28–51] years, *p* = 0.820), sex (61.0% vs. 59.0% male, *p* = 0.905), weight (82.1 ± 10.3 vs. 84.3 ± 7.5 kg, *p* = 0.098), and BMI (24.76 ± 3.01 vs. 24.64 ± 2.35 kg/m^2^, *p* = 0.761). Smoking was reported by 23 of 100 IBD patients (23.0%) and 16 of 78 controls (20.5%) (*p* = 0.829). IBD patients were significantly shorter (1.80 [1.75–1.87] vs. 1.85 [1.80–1.90] m, *p* = 0.006).

Compared to controls, IBD patients exhibited significantly lower haemoglobin (136.5 vs. 151 g/L, *p* < 0.001), haematocrit (0.42 vs. 0.46 L/L, *p* < 0.001), serum iron (15 vs. 18 µmol/L, *p* = 0.013), ferritin (31.5 vs. 73 µg/L, *p* = 0.001), albumin (40 vs. 44 g/L, *p* < 0.001), cholesterol (4.60 vs. 5.15 mmol/L, *p* = 0.032), and LDL (2.80 vs. 3.10 mmol/L, *p* = 0.014). IBD patients had significantly higher hs-CRP (2.60 vs. 1.00 mg/L, *p* < 0.001) and platelet counts (260 vs. 229 × 10^9^/L, *p* < 0.001). Demographic and biochemical characteristics are presented in Table 1.

**Table 1:** Demographic and biochemical characteristics: IBD patients vs. healthy controls.

Variable	IBD (n = 100)	Controls (n = 78)	*p*-Value ^†^
Age (years)	39 [28–47]	37 [28–51]	0.820
Sex, male (%)	61.0%	59.0%	0.905 ^§^
Smoking, yes, n (%)	23 (23.0%)	16 (20.5%)	0.829 ^§^
Weight (kg)	82.1 ± 10.3	84.3 ± 7.5	0.098 ^‡^
Height (m)	1.8 [1.75–1.87]	1.85 [1.8–1.9]	0.006
BMI (kg/m^2^)	24.76 ± 3.01	24.64 ± 2.35	0.761 ^‡^
Haemoglobin (g/L)	136.5 [128–153]	151 [140–157]	<0.001
Haematocrit (L/L)	0.42 [0.39–0.46]	0.46 [0.42–0.47]	<0.001
Platelets (×10^9^/L)	260 [226–297]	229 [200.2–260.2]	<0.001
Albumin (g/L)	40 [37–42]	44 [42–45]	<0.001
hs-CRP (mg/L)	2.60 [0.85–15.62]	1.00 [0.43–1.80]	<0.001
Iron (µmol/L)	15 [7–21]	18 [14.2–20.8]	0.013
Ferritin (µg/L)	31.5 [12.8–73]	73 [26.5–126.5]	0.001
Cholesterol (mmol/L)	4.6 [3.85–5.7]	5.15 [4.33–5.8]	0.032
LDL (mmol/L)	2.8 [2.1–3.6]	3.1 [2.42–3.88]	0.014
HDL (mmol/L)	1.3 [1–1.6]	1.3 [1.2–1.6]	0.212
Triglycerides (mmol/L)	0.95 [0.8–1.42]	1 [0.7–1.77]	0.925
ALT (U/L)	19 [13–26.2]	22.5 [16–32]	0.025
Urea (mmol/L)	4.35 [3.58–5.4]	5.3 [4.5–6.2]	<0.001
Creatinine (µmol/L)	70.5 [63.8–76]	74 [67–85]	0.006
Uric acid (µmol/L)	261 [211–303.8]	302.5 [242.5–341]	0.004

Note: Data are presented as median [IQR], except weight and BMI, which are presented as mean ± SD. Test symbols: ^†^ Mann–Whitney U test; ^‡^ Welch’s independent-samples *t*-test; ^§^ χ^2^ test with continuity correction. Abbreviations: IBD, inflammatory bowel disease; BMI, body mass index; SD, standard deviation; IQR, interquartile range; hs-CRP, high-sensitivity C-reactive protein; LDL, low-density lipoprotein cholesterol; HDL, high-density lipoprotein cholesterol; ALT, alanine aminotransferase.

### 3.2. IBD Clinical Characteristics

Clinical characteristics of IBD patients stratified by disease subtype are presented in Table 2. Disease duration was comparable between UC (9.0 [5.0–11.5] years) and CD (6.2 [3.0–11.5] years; *p* = 0.280). The proportion with score-defined active disease was high in both subtypes: 43 of 51 UC patients (84%; MES ≥ 2) and 45 of 49 CD patients (92%; SES-CD ≥ 3; *p* = 0.232). Among UC patients, median UCEIS was 6.0 [5.0–7.8] and MES was 3.0 [2.0–3.0]. For CD, CDAI was 57.0 [35.0–97.0]; HBI median was 2.0 [1.0–4.0]; SES-CD was 12.0 [6.6–19.8]. Surgery history was significantly more frequent in CD (33%) than UC (2%; *p* < 0.001). Biological therapy was the most common treatment in both subtypes (UC: 57%, CD: 67%; *p* = 0.280). Faecal calprotectin and hs-CRP were similar between subtypes. IBDQ-32 total score was lower in CD than in UC in the complete time-dependent IBDQ-32 dataset (*p* = 0.030; Table 2).

In an additional exploratory comparison of the 20 demographic and biochemical variables presented in Table 1, no UC–CD difference remained significant after BH-FDR correction (all q ≥ 0.165; Supplementary Table S11). Albumin was nominally lower in UC than in CD (39 [35.5–42] vs. 41 [39–42] g/L; unadjusted *p* = 0.008, q = 0.165); none of the other 19 Table 1 variables showed an unadjusted between-subtype difference (all *p* ≥ 0.058).

### 3.3. Sampling Timing and Analytical Retention

In the time-dependent analytical sample, wake time did not differ between groups (IBD: 445 [405–480] min vs. controls: 435 [403–480] min; *p* = 0.941). After exclusion of three IBD records with invalid C0-to-C1 timing, the C0-to-C1 sampling interval was comparable between groups (IBD: 34 [30–35] min vs. controls: 35 [31–35] min; *p* = 0.201). In a post hoc sensitivity analysis restricted to participants with a recorded C0-to-C1 interval of 30–40 min, the between-group findings remained materially unchanged. Patients with IBD continued to have lower C0 (0.47 vs. 1.07 µg/dL), C1 (0.90 vs. 1.96 µg/dL), ΔCAR (0.14 vs. 0.48 µg/dL), and CAR-specific AUCi (2.1 vs. 8.8 min·µg/dL), with all comparisons remaining statistically significant at *p* < 0.001.

### 3.4. Salivary Cortisol Concentrations

**Figure 2:**
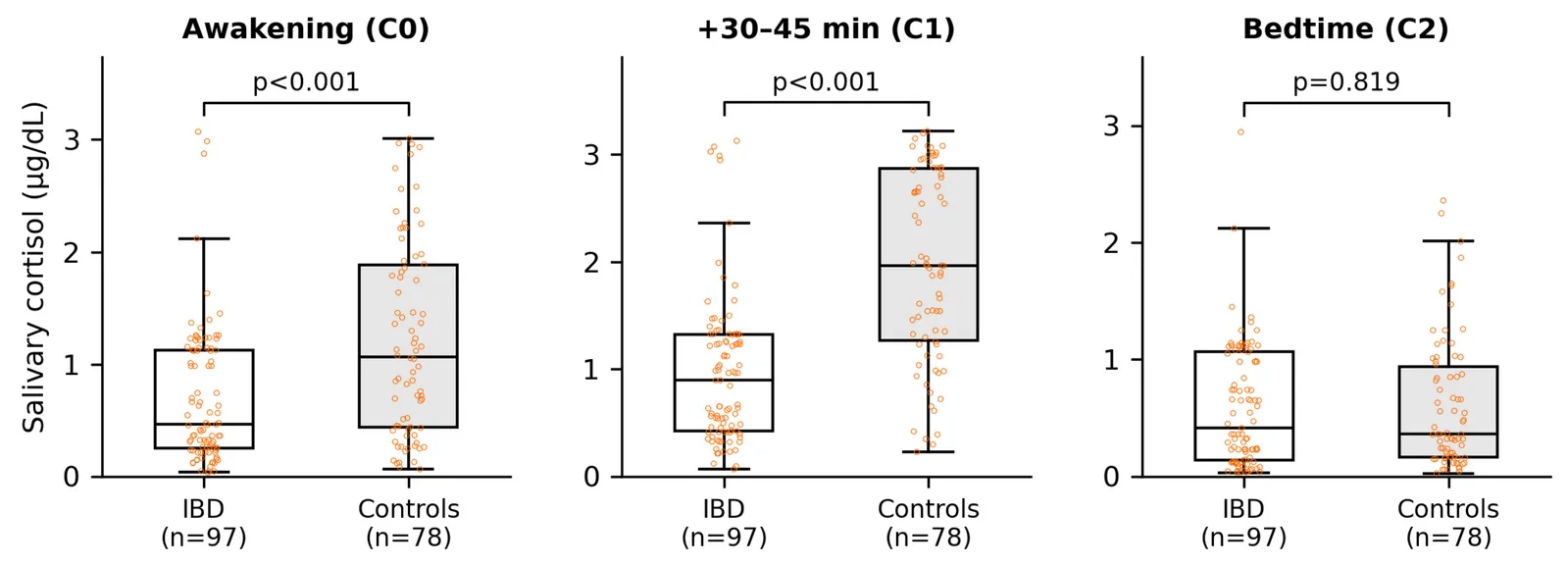
Salivary cortisol concentrations (µg/dL) at awakening (C0), +30–45 min post-awakening (C1), and bedtime (C2) in IBD patients and healthy controls. Data are box plots showing median, IQR, and 1.5 × IQR whiskers. Comparisons were performed using the Mann–Whitney U test.

Salivary cortisol concentrations at the three time points are shown in Figure 2 for the time-dependent analytical sample. IBD patients exhibited markedly lower awakening cortisol (C0: 0.478 [0.258–1.125] µg/dL vs. controls: 1.067 [0.436–1.881] µg/dL; *p* < 0.001) and substantially lower post-awakening cortisol (C1: 0.850 [0.412–1.321] vs. 1.950 [1.124–2.844] µg/dL; *p* < 0.001). Evening cortisol (C2) did not differ significantly between groups (IBD: 0.415 [0.137–1.064] vs. controls: 0.360 [0.163–0.939] µg/dL; *p* = 0.819).

### 3.5. Cortisol Awakening Response and Time-Dependent Cortisol Metrics

**Figure 3:**
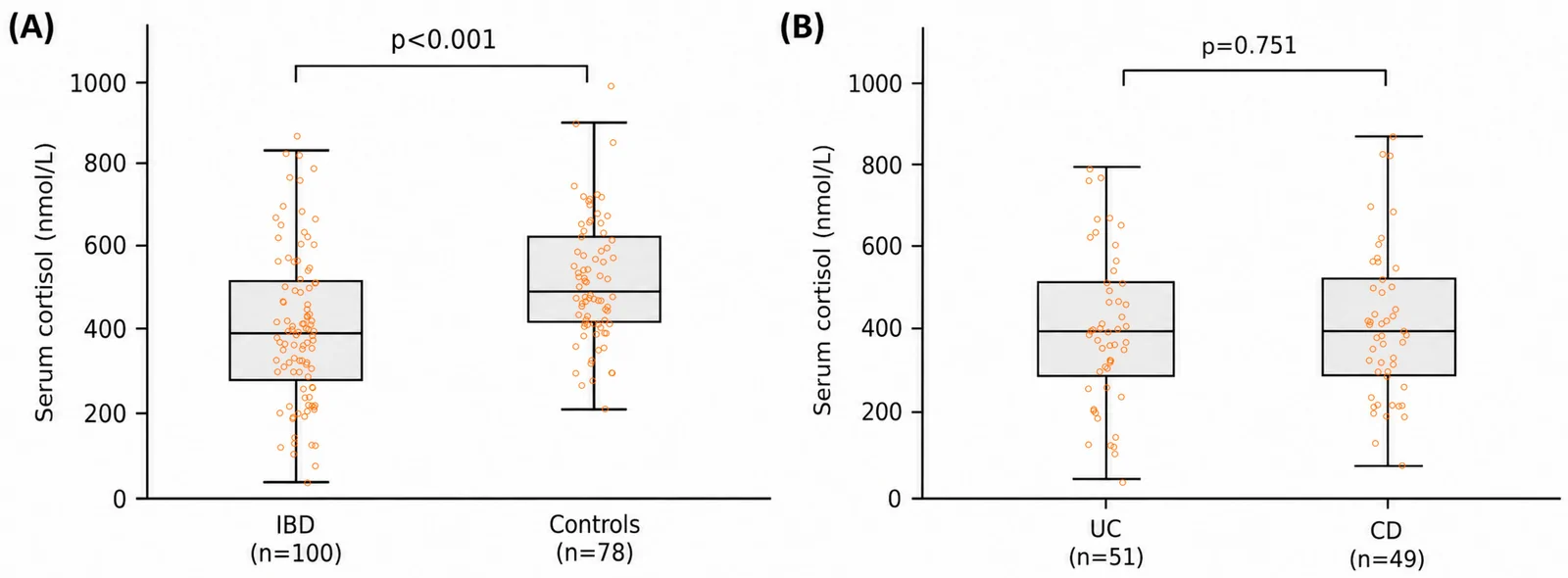
Serum cortisol concentrations (nmol/L) in the full enrolled cohort. (**A**) Comparison between patients with inflammatory bowel disease (IBD; n = 100) and healthy controls (n = 78). (**B**) Comparison between patients with ulcerative colitis (UC; n = 51) and Crohn’s disease (CD; n = 49). Boxes represent medians and interquartile ranges (IQRs), with whiskers extending to 1.5 × IQR. Individual observations and outlier markers are not displayed. Between-group comparisons were performed using the Mann–Whitney U test; exact *p*-values are shown above the panels.

All CAR-specific metrics were substantially attenuated in IBD patients compared to healthy controls (Table 3). The ΔCAR was markedly lower in IBD (0.130 [0.069–0.261] vs. 0.461 [0.192–0.865] µg/dL; *p* < 0.001). CAR-specific AUCi (computed over the C0→C1 window) was substantially lower in IBD (2.080 [1.062–4.025] vs. 7.770 [3.259–13.976] min·µg/dL; *p* < 0.001), reflecting reduced integrated cortisol output during the immediate post-awakening period. CAR-specific AUCg (total C0→C1 trapezoidal area) was also significantly lower (20.0 [11.5–38.6] vs. 52.2 [33.9–69.1] min·µg/dL; *p* < 0.001). Partial waking-to-bed AUCg, incorporating all three time points, was substantially lower in IBD (517 [281–1056] vs. 1131 [654–1516] min·µg/dL; *p* < 0.001), consistent with reduced sparse-sampling waking-day cortisol exposure largely reflecting lower morning cortisol concentrations. Daily amplitude (C1 − C2), relative CAR, and C1/C2 ratio were all significantly reduced in IBD. The post-awakening-to-bedtime diurnal cortisol slope (DCS) was significantly flatter in IBD, with less negative values than controls (−0.031 [−0.073 to −0.017] vs. −0.116 [−0.241 to −0.054] log_2_ µg/dL/hour; *p* < 0.001). These findings are summarised in Table 3. Serum cortisol measured in the full enrolled cohort was lower in IBD than in controls (387.5 [281.0–505.6] vs. 481.4 [414.2–610.8] nmol/L; *p* < 0.001). By contrast, ACTH and CBG did not differ significantly between groups. ACTH concentrations were comparable between IBD patients and controls (18.6 [12.4–27.8] vs. 20.4 [14.1–29.7] pg/mL; *p* = 0.347), as were CBG concentrations (36.1 [31.2–42.0] vs. 36.8 [32.0–43.6] mg/L; *p* = 0.596). Serum cortisol concentrations are shown in Figure 3A.

### 3.6. Multivariable Regression Analysis of Time-Dependent Cortisol Outcomes

Multivariable and sensitivity analyses supported the robustness of the between-group cortisol differences (Table 4). In natural-log-transformed OLS models with HC3 heteroscedasticity-consistent standard errors, IBD remained associated with lower awakening cortisol (C0 IBD/control ratio = 0.530, 95% CI 0.399–0.706, *p* < 0.001) and lower post-awakening cortisol (C1 ratio = 0.426, 95% CI 0.345–0.528, *p* < 0.001), whereas bedtime cortisol was not clearly different between groups (C2 ratio = 0.963, 95% CI 0.683–1.359, *p* = 0.830). IBD was also associated with lower CAR-specific AUCi among positive AUCi records (ratio = 0.271, 95% CI 0.189–0.388, *p* < 0.001), lower CAR-specific AUCg (ratio = 0.453, 95% CI 0.363–0.565, *p* < 0.001), and lower partial waking-to-bed AUCg (ratio = 0.516, 95% CI 0.415–0.642, *p* < 0.001).

To determine whether the lower post-awakening C1 concentration reflected only lower awakening cortisol, a residualised post-awakening model was fitted with log(C1) as the outcome and log(C0) included as an additional covariate. IBD remained independently associated with lower post-awakening cortisol after adjustment for C0, age, sex, BMI, wake time, and dt1 (IBD/control ratio = 0.606, 95% CI 0.506–0.725, *p* < 0.001). Thus, the attenuated post-awakening cortisol profile in IBD was not explained solely by lower awakening cortisol.

In a repeated-measures mixed model including all three salivary cortisol time points, the IBD-by-time-point interaction was significant (Wald χ^2^[2] = 36.34, *p* = 1.29 × 10^−8^), indicating that the group difference varied across the sampling day. Estimated IBD/control ratios were 0.546 for C0 (95% CI 0.413–0.723, *p* < 0.001), 0.431 for C1 (95% CI 0.325–0.570, *p* < 0.001), and 0.925 for C2 (95% CI 0.699–1.225, *p* = 0.588). This model confirmed that the IBD-associated cortisol difference was concentrated in the awakening and post-awakening period, with no clear adjusted difference at bedtime.

Median quantile regression yielded the same overall pattern, with lower adjusted medians for C0, C1, ΔCAR, CAR-specific AUCi, CAR-specific AUCg, and partial waking-to-bed AUCg and no clear adjusted difference in C2. Raw-scale HC3 OLS estimates also remained significant in the same direction for ΔCAR, CAR-specific AUCi, CAR-specific AUCg, and partial waking-to-bed AUCg. Full sensitivity-model estimates are provided in Supplementary Tables S8–S10.

### 3.7. Disease Phenotype, Inflammatory Activity, and Cortisol Dynamics

To determine whether the attenuated cortisol profile in IBD was explained by disease subtype or score-defined disease activity, subgroup comparisons and continuous correlation analyses were performed across inflammatory and clinical activity markers.

Patients with UC (n = 49) and CD (n = 48) did not differ significantly on any salivary cortisol-derived time-dependent measure: ΔCAR (*p* = 0.631), CAR-specific AUCi (*p* = 0.621), partial waking-to-bed AUCg (*p* = 0.601), and DCS (*p* = 0.911) were all comparable between subtypes, as were individual salivary time-point concentrations (C0, C1, C2; all *p* ≥ 0.46). Full time-dependent subgroup data are provided in Supplementary Table S1. In the full IBD cohort, serum cortisol concentrations were also comparable between UC and CD patients (387.9 [279.9–502.9] vs. 387.2 [287.3–503.5] nmol/L; *p* = 0.751; Figure 3B).

Within the IBD time-dependent analytical sample, score-defined disease activity was classified using subtype-specific endoscopic score thresholds: MES ≥ 2 for UC and SES-CD ≥ 3 for CD. Among IBD patients who satisfied the sampling-time criteria for time-dependent analyses, this identified 86 patients with score-defined active disease and 11 in score-defined remission. No consistent differences were observed between score-defined active disease and remission on cortisol outcomes. Among 86 patients with active disease and 11 in remission, C0 was 0.547 [0.258–1.147] versus 0.365 [0.142–0.415] µg/dL (*p* = 0.050; r_rb = 0.36, 95% CI 0.00 to 0.73). The effect sizes were r_rb = 0.02 (95% CI −0.34 to 0.39) for ΔCAR, r_rb = 0.02 (95% CI −0.35 to 0.38) for CAR-specific AUCi, r_rb = 0.36 (95% CI −0.01 to 0.72) for CAR-specific AUCg, r_rb = 0.35 (95% CI −0.01 to 0.72) for partial waking-to-bed AUCg, r_rb = 0.29 (95% CI −0.08 to 0.65) for DCS, and r_rb = −0.01 (95% CI −0.38 to 0.35) for serum cortisol. Full results are provided in Supplementary Table S2.

Spearman correlation analyses within the IBD time-dependent analytical sample (n = 97) were corrected for multiple comparisons using a global BH-FDR correction across a 140-test biomarker-cortisol matrix. hs-CRP was the most consistent inflammatory correlate of altered morning cortisol dynamics. Higher hs-CRP was positively associated with awakening cortisol C0 (ρ = +0.325, *p* = 0.001, q = 0.041) and inversely associated with CAR-derived reactivity measures, including ΔCAR (ρ = −0.402, *p* < 0.001, q = 0.002), CAR-specific AUCi (ρ = −0.408, *p* < 0.001, q = 0.002), and relative CAR (ρ = −0.517, *p* < 0.001, q < 0.001). CAR-specific AUCg showed a nominal positive association with hs-CRP (ρ = +0.238, *p* = 0.019) that did not survive global FDR correction. The hs-CRP–cortisol associations that remained significant after global BH-FDR correction are summarized in Table 5.

Among other inflammatory biomarkers, faecal calprotectin showed within-table FDR-significant but not globally FDR-significant associations with C0 (ρ = +0.268, *p* = 0.008) and relative CAR (ρ = −0.280, *p* = 0.005). Nominal positive associations were also observed with C2, CAR-specific AUCg, and partial waking-to-bed AUCg, but none survived global FDR correction. Platelet count showed nominal inverse associations with ΔCAR and CAR-specific AUCi in the broader biomarker matrix, also not surviving global FDR correction. No robust FDR-corrected associations were observed between albumin, haemoglobin, and any cortisol metric. Overall, these analyses suggest that the reduced morning cortisol profile was not clearly explained by IBD subtype or endoscopic score-defined activity category, while hs-CRP showed the most consistent relationship with altered CAR-derived reactivity. Selected correlation results are provided in Supplementary Tables S3 and S4.

Subtype-specific endoscopic-score analyses did not identify robust associations between endoscopic severity and cortisol outcomes. Within the UC subgroup, MES was not significantly associated with any cortisol parameter after within-table BH-FDR correction; the strongest unadjusted association was observed for relative CAR (ρ = −0.197, *p* = 0.180), but this did not survive correction and should be interpreted as exploratory. Similarly, within the CD subgroup, SES-CD was not significantly associated with any cortisol outcome after within-table BH-FDR correction. The strongest unadjusted associations were observed for C1/C2 ratio (ρ = −0.209, *p* = 0.155), CAR-specific AUCi (ρ = −0.186, *p* = 0.205), and ΔCAR (ρ = −0.178, *p* = 0.225), none of which reached statistical significance. These subtype-specific analyses should be interpreted cautiously, particularly for MES, which is an ordinal endoscopic score with a limited 0–3 range. Detailed results are provided in Supplementary Tables S5 and S6.

Cortisol parameters were compared according to current biological therapy status in exploratory, unadjusted analyses. No clear differences were observed for serum cortisol or the main salivary cortisol outcomes. Daily amplitude was nominally lower among patients receiving biological therapy than among those not receiving biological therapy (0.205 [0.094–0.327] vs. 0.246 [0.162–0.501] µg/dL, *p* = 0.044), but this exploratory finding should not be interpreted as a treatment effect because analyses were not adjusted for treatment indication, disease severity, cumulative steroid exposure, or prior treatment history. Detailed results are provided in Supplementary Table S7.

### 3.8. Exploratory IBDQ-32 Correlations with Cortisol Parameters

IBDQ-32 total score was available for the 97 IBD patients included in the time-dependent analytical sample. In exploratory analyses, higher IBDQ-32 total score, reflecting better disease-related QoL, was inversely associated with several absolute cortisol-output measures, whereas associations with CAR-derived reactivity metrics were weak (Table 6).

The strongest association was observed for awakening salivary cortisol C0 (ρ = −0.309, *p* = 0.002). C1, CAR-specific AUCg, partial waking-to-bed AUCg, daily amplitude, and serum cortisol showed nominal associations, whereas ΔCAR, CAR-specific AUCi, relative CAR, and C1/C2 ratio were not associated with IBDQ-32 total score. Overall, the exploratory IBDQ-32 pattern suggests that disease-related QoL may relate more closely to absolute morning cortisol output than to CAR-derived reactivity.

## 4. Discussion

In this frequency-matched case–control study, established IBD was associated with a coherent attenuation of early-morning HPA-axis dynamics. Patients with IBD had lower awakening and post-awakening salivary cortisol, reduced ΔCAR, CAR-specific AUCi and AUCg, lower sparse-sampling waking-day AUCg, a flatter post-awakening-to-bedtime cortisol slope, and lower next-morning fasting serum cortisol, whereas bedtime salivary cortisol, ACTH, and CBG did not differ. These findings were robust to log-transformed HC3 models, median quantile regression, and repeated-measures mixed modelling, and the lower C1 concentration persisted after adjustment for C0. Thus, the principal signal is not simply a single low morning cortisol value but an attenuated early-day cortisol phenotype in IBD.

A contemporary interpretation of this phenotype requires caution about what CAR represents. CAR is not a generic index of momentary psychological stress but a tightly timed post-awakening cortisol profile influenced by circadian phase, sleep–wake transitions, expected day demands, environmental context, and neurocognitive regulation [23,24,25]. Recent high-resolution interstitial cortisol data further challenge the assumption that awakening necessarily triggers a discrete burst of cortisol secretion, suggesting that the post-awakening rise may partly reflect the continuation of pre-awakening circadian dynamics [27]. Accordingly, the present findings should be framed as evidence of altered morning cortisol dynamics in IBD, not as direct proof of impaired acute stress reactivity or clinical adrenal insufficiency. This distinction is important because endogenous cortisol availability, glucocorticoid-receptor sensitivity, tissue-specific glucocorticoid signalling, and pharmacological glucocorticoid therapy are not interchangeable concepts [46,47].

The results extend limited human evidence on HPA-axis perturbation in IBD. Earlier work suggested uncoupling between sympathetic and HPA-axis activity in IBD [28], while a prospective CD cohort reported blunted CAR in patients in clinical remission in association with cognitive and inflammatory alterations [29]. A recent UC pilot study linked morning salivary cortisol to anxiety symptom severity but did not identify clear associations with CRP, faecal calprotectin, or endoscopic activity [30]. Compared with these studies, the present analysis adds a larger mixed IBD cohort, frequency-matched controls, recorded sampling intervals, CAR-derived metrics, fasting serum cortisol, endoscopic classification, inflammatory biomarkers, IBDQ-32 assessment, and multiplicity-controlled biomarker correlations. This strengthens the argument that morning HPA-axis attenuation is a reproducible candidate feature of IBD, while also showing that this feature cannot be reduced to IBD subtype or to a single endoscopic activity category.

Mechanistically, a blunted morning cortisol profile fits current gut–brain–immune models more plausibly than a simple chronic-stress-equals-hypercortisolism model. IBD is increasingly being conceptualised as a systemic disorder involving mucosal immune activation, epithelial barrier dysfunction, microbial and metabolic perturbation, autonomic and enteric nervous system signalling, and central stress-regulatory circuits [9,10,11,12,14,48,49,50]. Experimental work has identified plausible routes by which psychological stress can aggravate intestinal inflammation: chronic stress can alter enteric glial and myeloid-cell states and increase colitis severity, while HPA-axis-driven glucocorticoid/corticosterone signalling can impair intestinal stem-cell function and epithelial renewal in colitis models [20,21]. These findings do not imply that the lower cortisol observed here caused mucosal inflammation. Rather, they support a framework in which persistent inflammation, stress exposure, sleep and behavioural factors, autonomic tone, and tissue-level glucocorticoid sensitivity may jointly reshape HPA-axis dynamics. Under such conditions, lower morning cortisol output can coexist with inflammatory activity, particularly if glucocorticoid signalling is mismatched to immune demand or if glucocorticoid-receptor sensitivity is altered [19,46]. ACTH and CBG did not differ between groups; however, single fasting measurements cannot exclude differences in pulsatile ACTH secretion, dynamic adrenal responsiveness, cortisol clearance, temporal variation in cortisol-binding capacity, or tissue-level glucocorticoid sensitivity.

The biomarker pattern in this study is clinically informative. hs-CRP showed the most robust relationship with cortisol dynamics: higher hs-CRP correlated positively with awakening cortisol but inversely with ΔCAR, CAR-specific AUCi, and relative CAR after global FDR correction. This suggests a dissociation between basal awakening cortisol and the capacity for additional post-awakening increase. In practical terms, a single morning cortisol concentration could miss the blunted dynamic component captured by CAR-derived metrics. The weaker and non-globally significant associations for faecal calprotectin, and the lack of robust correlations with MES or SES-CD, suggest that CAR-derived metrics may reflect a systemic neuroendocrine-inflammatory layer rather than the same disease information captured by stool biomarkers or endoscopic mucosal activity. Because faecal calprotectin data were available for only 10 controls, this subset was insufficient for a valid between-group comparison or for establishing a stable healthy reference distribution. This limitation does not affect calculation of the correlations within the IBD cohort, but it prevents determining whether the observed calprotectin–cortisol associations extend into the healthy range or are specific to IBD. This interpretation is consistent with current treat-to-target and monitoring frameworks that emphasise symptoms, CRP, faecal calprotectin, endoscopic healing, histology, cross-sectional imaging, and intestinal ultrasound but do not currently include neuroendocrine markers in routine IBD monitoring [7,51,52]. CAR should therefore be positioned as a research biomarker candidate rather than as a clinical disease-activity measure.

The absence of clear UC-CD differences in cortisol measures also deserves a conservative interpretation. It suggests that early-morning HPA-axis attenuation may be a transdiagnostic IBD feature, but the study was not powered to prove equivalence between subtypes. The active disease-versus-remission comparison was underpowered and exploratory, with 86 patients with active disease and 11 in remission. The confidence intervals did not exclude activity-related differences or the possibility that CAR attenuation was concentrated in active disease. Cross-sectional activity categories may also be insufficient to capture cumulative inflammatory burden, recent flare history, sleep disruption, pain, fatigue, prior corticosteroid exposure, or treatment-selection effects. Longitudinal modelling will be needed to determine whether CAR metrics track within-person changes in inflammation, predict relapse, or identify patients with persistent fatigue and impaired QoL despite conventional biomarker control.

UC and CD were also broadly similar across the 20 demographic and biochemical variables evaluated in this additional exploratory analysis. Albumin was nominally lower in UC than in CD (39 [35.5–42] vs. 41 [39–42] g/L; unadjusted *p* = 0.008), but this difference did not remain significant after correction across 20 comparisons (q = 0.165). Neither hs-CRP nor faecal calprotectin differed by subtype. This finding should therefore be regarded as hypothesis-generating rather than evidence of a subtype-specific biochemical phenotype. The lack of FDR-significant differences in these 20 variables does not establish equivalence or exclude residual confounding; notably, surgery history differed substantially between subtypes. Because information on resection location and extent, postoperative nutritional consequences, and bile-acid disturbances was not sufficiently detailed, the present study could not determine whether previous surgery contributed to subtype-related HPA-axis variation.

The exploratory IBDQ-32 findings add a further layer. Better disease-related QoL was inversely associated mainly with absolute cortisol-output measures, especially C0, whereas CAR-reactivity indices showed weak associations. This pattern does not support a simplistic “more cortisol is better” interpretation. It may instead indicate that absolute morning cortisol is influenced by broader illness burden, sleep, pain, fatigue, psychological distress, or behavioural activation, while the CAR increment captures a different aspect of morning HPA-axis regulation. Recent reviews and intervention syntheses emphasise that anxiety, depression, fatigue, and stress-related processes are clinically important in IBD, but their biological relationships with objective inflammation are bidirectional and heterogeneous [13,14,15,48,49,50,53]. Therefore, future studies should assess CAR together with validated anxiety, depression, perceived-stress, sleep, autonomic, inflammatory, microbial, endoscopic/imaging, and patient-reported outcome measures, rather than treating cortisol as a stand-alone stress marker.

Strengths include consecutive patient recruitment, frequency-matched controls, exclusion of corticosteroid and oral-contraceptive exposures likely to confound cortisol assessment, standardised sampling instructions, prespecified timing-validity rules, duplicate salivary assays, blinded laboratory processing, use of actual sampling intervals in CAR calculations, adjusted and sensitivity analyses, and FDR-controlled biomarker testing. The lower next-morning fasting serum cortisol was directionally consistent with the salivary morning findings at the group level; however, because serum was obtained on a different day and reflects total rather than salivary free cortisol, it cannot be interpreted as time-matched salivary–serum concordance or as methodological validation of the salivary measurements.

Limitations remain substantial. The cross-sectional design prevents causal inference. At-home saliva collection relied on participant logs without electronic monitoring or actigraphy, which is particularly important because mistimed awakening samples can bias CAR estimates. Electronically monitored studies indicate that sampling delays can bias CAR estimates in either direction depending on their magnitude and position within the nonlinear post-awakening cortisol trajectory, while larger delays generally attenuate the estimated CAR [24,25]. Because ΔCAR is calculated as C1 − C0, any timing error that increases C0 or decreases C1 attenuates ΔCAR, whereas an error that decreases C0 or increases C1 inflates it; an isolated error of 0.10 µg/dL in either sample changes ΔCAR by 0.10 µg/dL. For CAR-specific AUCi, calculated as 0.5 × ΔCAR × dt1 [26], a 5 min interval error around the observed 35 min interval changes the time component by approximately 14%, even if the measured concentrations remain unchanged. Restriction according to the recorded C0-to-C1 interval assesses only inter-sample spacing and cannot exclude a common delay of both samples relative to true awakening. The protocol included only one day of sampling and one post-awakening sample, limiting assessment of CAR peak timing, day-to-day reliability, ultradian rhythmicity, and the full diurnal profile. Single-day CAR assessment has limited trait-level reliability because a substantial proportion of its variability is state- and situation-dependent [24,25]. Non-differential day-to-day variability would be expected primarily to increase within-group variance, widen confidence intervals, reduce statistical power, and limit reproducibility, whereas systematic between-group differences in nocturnal symptoms, sleep disturbance, or day-specific disease burden could bias the comparison in either direction. Future studies should assess CAR on at least two, and preferably three or more, separate sampling days [24,25]. The C1-to-C2 slope is therefore a sparse post-awakening-to-bedtime slope rather than a complete diurnal cortisol rhythm. Serum cortisol, ACTH, and CBG were measured the following morning rather than simultaneously with salivary samples, and DHEA-S, cytokine panels, hair cortisol, sleep metrics, autonomic measures, microbiome, and metabolomic data were not collected. Accordingly, these serum measurements provide only complementary next-day information and cannot validate the salivary C0/C1 profile or characterise pulsatile or stimulus-dependent HPA-axis activity. Subgroup analyses by IBD subtype, remission status, and biological therapy were exploratory and underpowered, and treatment analyses were not adjusted for indication, disease severity, cumulative corticosteroid history, or prior treatment exposure. Validated sleep quality was not assessed, and the PHQ-9 was not used as a substitute because it evaluates depressive symptoms rather than sleep quality; residual confounding by sleep disturbance, nocturnal symptoms, fatigue, and atypical sleep schedules therefore remains possible. Smoking was not included in the prespecified multivariable models because the adjustment set was defined a priori to account for the principal demographic and sampling-timing determinants of the CAR, and the study was not designed to estimate an independent smoking effect. Although the observed smoking prevalence differed only modestly between groups, this descriptive finding does not establish equivalence or exclude residual confounding by smoking. IBD-related surgery and detailed medication classes, including 5-aminosalicylates and immunomodulators, were not entered into the primary IBD-versus-control models because these were disease-specific exposures closely linked to disease subtype, duration, severity, and treatment indication. The study was not powered to estimate their independent associations with cortisol, and residual surgical and medication-related confounding remains possible. Thus, current or recent corticosteroid treatment is unlikely to account for the observed cortisol differences; however, cumulative or more remote corticosteroid exposure was not quantified, and residual confounding cannot be excluded. Finally, the single-centre design and the high proportion of score-defined active disease may limit generalisability to deeply remitted, newly diagnosed, paediatric, older, or population-based IBD cohorts.

In conclusion, established IBD was associated with a robustly attenuated early-morning cortisol phenotype characterised by lower awakening and post-awakening salivary cortisol, reduced CAR-derived metrics, lower partial waking-day cortisol exposure, a flatter sparse post-awakening-to-bedtime slope, and lower next-morning fasting serum cortisol. The pattern was not clearly subtype- or endoscopy-category-specific, while hs-CRP showed the strongest association with blunted post-awakening cortisol dynamics. These findings support CAR-derived metrics as candidate neuroendocrine markers within a gut–brain–immune model of IBD. Their clinical relevance now requires prospective, multi-day studies with objective sampling adherence, sleep and autonomic monitoring, inflammatory and microbial profiling, and longitudinal outcomes including relapse, treatment response, fatigue, and QoL.

## 5. Conclusions

In this cross-sectional case–control study, patients with IBD exhibited a markedly attenuated cortisol awakening response, reflected by lower awakening and post-awakening salivary cortisol concentrations and reduced ΔCAR and CAR-specific AUC measures. Bedtime salivary cortisol remained comparable between groups, while adjusted and sensitivity analyses confirmed that the differences were concentrated in the early-morning period. Cortisol dynamics did not differ consistently between UC and CD. The score-defined activity analysis was underpowered because only 11 patients were in remission, and activity-related differences in CAR cannot be excluded. The associations between higher hs-CRP and attenuated CAR-derived reactivity suggest a relationship between systemic inflammation and altered early-morning HPA-axis regulation. However, the cross-sectional design, sparse salivary sampling, and reliance on participant-recorded collection times preclude causal interpretation. These findings should therefore not be considered evidence of adrenal insufficiency but rather of altered morning cortisol dynamics in IBD. Longitudinal studies incorporating repeated multi-day sampling and objective adherence monitoring are needed to determine the stability, clinical significance, and treatment responsiveness of this neuroendocrine profile. The practical take-home message is that CAR should be regarded as an investigational biomarker: it may capture dynamic HPA-axis alterations missed by a single morning cortisol measurement, but it is not yet suitable for routine IBD monitoring or treatment decisions.

## Figures and Tables

**Table 2:** Clinical characteristics of IBD patients by disease subtype.

Parameter	UC (n = 51)	CD (n = 49)	*p*-Value ^†^
**General**			
Disease duration (years)	9.0 [5.0–11.5]	6.2 [3.0–11.5]	0.280
Score-defined active disease, n (%)	43 (84%)	45 (92%)	0.232 ^‡^
Surgery history, n (%)	1 (2%)	16 (33%)	<0.001 ^§^
**UC Disease Activity Indices (UC only)**			
UCEIS, median [IQR]	6.0 [5.0–7.8]	N/A	—
MES, median [IQR]	3.0 [2.0–3.0]	N/A	—
**CD Activity Indices (CD only)**			
CDAI Score, median [IQR]	N/A	57.0 [35.0–97.0]	—
Harvey-Bradshaw Index, median [IQR]	N/A	2.0 [1.0–4.0]	—
SES-CD, median [IQR]	N/A	12.0 [6.6–19.8]	—
**IBD Biomarkers**			
hs-CRP (mg/L)	3.6 [0.7–12.1]	2.5 [0.9–18.0]	0.748
Faecal calprotectin (µg/g)	147 [40–507]	147 [50–393]	0.929
**Patient-Reported Outcome**			
IBDQ-32 total score, median [IQR]	186 [169–202]	171 [141–196]	0.030
**Treatment**			
Biological therapy, n (%)	29 (57%)	33 (67%)	0.280 ^‡^
Anti-TNFα	21 (72%)	25 (76%)	0.670 ^‡^
Anti-integrin	5 (17%)	5 (15%)	0.890 ^§^
Other	3 (10%)	3 (9%)	0.990 ^§^
DMARD, n (%)	9 (18%)	7 (14%)	0.647 ^‡^
Aminosalicylates, n (%)	13 (25%)	9 (18%)	0.390 ^‡^

Note: Data are presented as median [IQR] or n (%), as appropriate. Test symbols: ^†^ Mann–Whitney U test; ^‡^ χ^2^ test; ^§^ Fisher’s exact test. Score-defined active disease was defined as MES ≥ 2 for UC and SES-CD ≥ 3 for CD. Abbreviations: UC, ulcerative colitis; CD, Crohn’s disease; IQR, interquartile range; UCEIS, Ulcerative Colitis Endoscopic Index of Severity; MES, Mayo endoscopic subscore; CDAI, Crohn’s Disease Activity Index; SES-CD, Simple Endoscopic Score for Crohn’s Disease; hs-CRP, high-sensitivity C-reactive protein; IBDQ-32, 32-item Inflammatory Bowel Disease Questionnaire; DMARD, disease-modifying antirheumatic drug; N/A, not applicable.

**Table 3:** Timing parameters, CAR metrics, and diurnal cortisol slope: IBD patients vs. healthy controls.

Parameter	IBD Median [IQR] (n = 97)	Controls Median [IQR] (n = 78)	*p*-Value ^†^
Wake time, T0 (min after midnight)	445 [405–480]	435 [403–480]	0.941
C0-to-C1 interval, dt1 (min)	34 [30–35]	35 [31–35]	0.201
ΔCAR (C1 − C0) (µg/dL)	0.130 [0.069–0.261]	0.461 [0.192–0.865]	<0.001
CAR-specific AUCi (min·µg/dL)	2.080 [1.062–4.025]	7.770 [3.259–13.976]	<0.001
CAR-specific AUCg (min·µg/dL)	20.0 [11.5–38.6]	52.2 [33.9–69.1]	<0.001
Partial waking-to-bed AUCg (min·µg/dL)	517 [281–1056]	1131 [654–1516]	<0.001
Daily amplitude C1 − C2 (µg/dL)	0.201 [0.098–0.330]	1.124 [0.612–1.879]	<0.001
Relative CAR (%)	26.9 [9.1–84.8]	37.4 [12.9–220.7]	0.045
C1/C2 ratio	1.391 [1.192–2.346]	3.403 [1.668–9.645]	<0.001
Diurnal cortisol slope, DCS (log_2_ µg/dL/hour)	−0.031 [−0.073 to −0.017]	−0.116 [−0.241 to −0.054]	<0.001
ACTH (pg/mL)	18.6 [12.4–27.8]	20.4 [14.1–29.7]	0.347
CBG (mg/L)	36.1 [31.2–42.0]	36.8 [32.0–43.6]	0.596

Note: Data are presented as median [IQR]. ^†^ Mann–Whitney U test. Abbreviations: IBD, inflammatory bowel disease; IQR, interquartile range; CAR, cortisol awakening response; C0, awakening salivary cortisol; C1, salivary cortisol at +30–45 min after awakening; C2, bedtime salivary cortisol; dt1, C0-to-C1 sampling interval; AUCi, area under the curve with respect to increase; AUCg, area under the curve with respect to ground; DCS, diurnal cortisol slope; ACTH, adrenocorticotropic hormone; CBG, corticosteroid-binding globulin.

**Table 4:** Robust multivariable models for time-dependent cortisol outcomes.

Model	Outcome	Effect Ratio Estimate for IBD	95% CI	*p*-Value
log-HC3 OLS	C0 awakening cortisol	0.530	0.399–0.706	<0.001
	C1 post-awakening cortisol	0.426	0.345–0.528	<0.001
	C2 bedtime cortisol	0.963	0.683–1.359	0.830
	C1 adjusted for C0	0.606	0.506–0.725	<0.001
	CAR-specific AUCi	0.271	0.189–0.388	<0.001
	CAR-specific AUCg	0.453	0.363–0.565	<0.001
	Partial waking-to-bed AUCg	0.516	0.415–0.642	<0.001
mixed model	C0 profile (mixed)	0.546	0.413–0.723	<0.001
	C1 profile (mixed)	0.431	0.325–0.570	<0.001
	C2 profile (mixed)	0.925	0.699–1.225	0.588

Note: Log-HC3 models used natural-log-transformed outcomes and HC3 heteroscedasticity-consistent standard errors; estimates are shown as exponentiated IBD/control ratios. Ratios below 1 indicate lower adjusted cortisol values or AUC-derived values in IBD patients compared with controls. Models were adjusted for age, sex, BMI, wake time, and dt1. The C1 adjusted-for-C0 model additionally included log(C0). The log(AUCi) model included positive AUCi records only (n = 174); other listed salivary models used the time-dependent analytical sample (n = 175). The mixed model included log-transformed salivary cortisol across C0, C1, and C2 with a participant-level random intercept and fixed effects for IBD status, time point, IBD × time-point interaction, age, sex, BMI, wake time, and dt1. AUCi log models were restricted to positive AUCi records. Abbreviations: OLS, ordinary least squares; HC3, heteroscedasticity-consistent standard errors; IBD, inflammatory bowel disease; CI, confidence interval; BMI, body mass index; CAR, cortisol awakening response; C0, awakening salivary cortisol; C1, salivary cortisol at +30–45 min after awakening; C2, bedtime salivary cortisol; dt1, C0-to-C1 sampling interval; AUCi, area under the curve with respect to increase; AUCg, area under the curve with respect to ground;

**Table 5:** Selected associations between hs-CRP and cortisol outcomes in patients with IBD.

Cortisol Outcome	Spearman ρ	Unadjusted *p*	q (Global BH-FDR)
C0: awakening salivary cortisol	+0.325	0.001	0.041
ΔCAR (C1 − C0)	−0.402	<0.001	0.002
CAR-specific AUCi	−0.408	<0.001	0.002
Relative CAR	−0.517	<0.001	<0.001

Note: Abbreviations: IBD, inflammatory bowel disease; hs-CRP, high-sensitivity C-reactive protein; BH-FDR, Benjamini–Hochberg false discovery rate; CAR, cortisol awakening response; C0, awakening salivary cortisol; C1, salivary cortisol at +30–45 min after awakening; AUCi, area under the curve with respect to increase; ρ, Spearman correlation coefficient.

**Table 6:** IBDQ-32 total score correlations with cortisol parameters in the IBD time-dependent analytical sample.

Cortisol Parameter	Spearman ρ	*p*-Value	q (BH-FDR)
C0: awakening salivary cortisol	−0.309	0.002	0.022
C1: +30–45 min salivary cortisol	−0.236	0.020	0.051
C2: bedtime salivary cortisol	−0.171	0.094	0.148
ΔCAR (C1 − C0)	−0.124	0.226	0.249
CAR-specific AUCi	−0.126	0.219	0.249
CAR-specific AUCg	−0.252	0.013	0.051
Partial waking-to-bed AUCg	−0.222	0.029	0.053
Daily amplitude (C1 − C2)	−0.237	0.019	0.051
Relative CAR	+0.152	0.137	0.188
C1/C2 ratio	+0.037	0.719	0.719
Serum cortisol	−0.231	0.023	0.051

Note: Abbreviations: IBDQ-32, 32-item Inflammatory Bowel Disease Questionnaire; BH-FDR, Benjamini–Hochberg false discovery rate; CAR, cortisol awakening response; C0, awakening salivary cortisol; C1, salivary cortisol at +30–45 min after awakening; C2, bedtime salivary cortisol; AUCi, area under the curve with respect to increase; AUCg, area under the curve with respect to ground; ρ, Spearman correlation coefficient.

## Data Availability

The data presented in this study are available upon request to the corresponding author. The data are not publicly available because some of the data sets will be used for further research.

## Supplementary Materials

The following supporting information can be downloaded at: https://www.mdpi.com/article/10.3390/medsci14040463/s1, Supplementary Tables S1–S11: Extended subgroup comparisons, selected biomarker correlations, sensitivity analyses, and exploratory UC–CD comparisons of the demographic and biochemical variables presented in Table 1.

## Abbreviations

The following abbreviations are used in this manuscript:

ACTH	Adrenocorticotropic hormone
ALT	Alanine aminotransferase
AUC	Area under the curve
AUCg	Area under the curve with respect to ground
AUCi	Area under the curve with respect to increase
BH-FDR	Benjamini–Hochberg correction for the false discovery rate
BMI	Body mass index
C0	Awakening salivary cortisol
C1	Salivary cortisol 30–45 min after awakening
C2	Bedtime salivary cortisol
CAR	Cortisol awakening response
ΔCAR	Absolute cortisol awakening response (C1 − C0)
CBG	Corticosteroid-binding globulin
CD	Crohn’s disease
CDAI	Crohn’s Disease Activity Index
CI	Confidence interval
CLIA	Chemiluminescence immunoassay
CRP	C-reactive protein
DCS	Diurnal cortisol slope
DMARD	Disease-modifying antirheumatic drug
dt1	C0-to-C1 sampling interval
dt2	C1-to-C2 sampling interval
ECCO	European Crohn’s and Colitis Organisation
ECLIA	Electrochemiluminescence immunoassay
ESGAR	European Society of Gastrointestinal and Abdominal Radiology
FDR	False discovery rate
HBI	Harvey-Bradshaw Index
HC3	Heteroscedasticity-consistent covariance matrix estimator, type 3
HDL	High-density lipoprotein cholesterol
HPA	Hypothalamic–pituitary–adrenal axis
hs-CRP	High-sensitivity C-reactive protein
IBD	Inflammatory bowel disease
IBDQ-32	32-item Inflammatory Bowel Disease Questionnaire
IQR	Interquartile range
LDL	Low-density lipoprotein cholesterol
MES	Mayo endoscopic subscore
OLS	Ordinary least squares
PHQ-9	9-item Patient Health Questionnaire
QoL	Quality of life
SD	Standard deviation
SES-CD	Simple Endoscopic Score for Crohn’s Disease
T0	Recorded awakening time
T1	Recorded post-awakening sampling time
T2	Recorded bedtime sampling time
UC	Ulcerative colitis
UCEIS	Ulcerative Colitis Endoscopic Index of Severity
