# Supplementary material for: Blunted Cortisol Awakening Response in Patients with Inflammatory Bowel Disease: A Cross-Sectional Case–Control Study
Source: Med Sci (Basel). 2026 Aug 7;14(4):463. doi: 10.3390/medsci14040463 (PMC13515125; doi:10.3390/medsci14040463)
Supplement: Supplementary file 1 [file medsci-14-00463-s001.zip › medsci-4480510-supplementary.pdf]

medical sciences

Blunted Cortisol Awakening Response in Patients with Inflammatory Bowel Disease: A Cross-Sectional Case–Control Study

Supplementary Table S1. UC vs. CD (Mann–Whitney U; n = 49 UC / 48 CD)

Cortisol outcome	UC, median [Q1-Q3] (n=49)	CD, median [Q1-Q3] (n=48)	p-value
C0 (μg/dL)	0.405 [0.265-1.125]	0.557 [0.249-1.149]	0.767
C1 (μg/dL)	0.896 [0.423-1.325]	0.847 [0.400-1.242]	0.662
C2 (μg/dL)	0.360 [0.230-1.080]	0.478 [0.098-1.001]	0.468
ΔCAR (μg/dL)	0.130 [0.084-0.234]	0.131 [0.062-0.283]	0.631
CAR-specific AUCi (min·μg/dL)	2.080 [1.302-3.622]	1.905 [0.938-4.494]	0.621
CAR-specific AUCg (min·μg/dL)	20.025 [11.655-38.940]	21.495 [11.476-36.159]	0.897
Partial waking-to-bed AUCg (min·μg/dL)	586.038 [301.710-1124.735]	491.740 [279.256-1030.213]	0.601
Relative CAR (%)	28.367 [10.032-84.800]	24.330 [6.972-86.629]	0.535
C1/C2 ratio	1.378 [1.192-1.997]	1.409 [1.202-2.695]	0.882
DCS ($\log_2$ μg/dL/hour)	−0.031 [−0.068 to −0.017]	−0.032 [−0.111 to −0.016]	0.911

Note: Values are presented as median [IQR]. Comparisons between UC and CD were performed using the Mann–Whitney U test in the IBD time-dependent analytical sample after exclusion of three non-analysable timing records. UC = ulcerative colitis; CD = Crohn's disease; CAR = cortisol awakening response; C0 = awakening salivary cortisol; C1 = salivary cortisol at +30-45 min after awakening; C2 = bedtime salivary cortisol; AUCi = area under the curve with respect to increase; AUCg = area under the curve with respect to ground; DCS = diurnal cortisol slope.

Supplementary Table S2. Score-defined active disease vs. remission (Mann–Whitney U; n = 86 active / 11 remission)

Cortisol outcome	Active, median [Q1-Q3] (n=86)	Remission, median [Q1-Q3] (n=11)	r_rb (95% CI)	p-value
C0 (µg/dL)	0.547 [0.258-1.147]	0.365 [0.142-0.415]	0.36 (0.00 to 0.73)	0.050
C1 (µg/dL)	0.965 [0.458-1.325]	0.412 [0.368-0.789]	0.31 (−0.06 to 0.67)	0.098
C2 (µg/dL)	0.540 [0.142-1.080]	0.230 [0.120-0.307]	0.30 (−0.07 to 0.66)	0.110
ΔCAR (µg/dL)	0.140 [0.081-0.240]	0.124 [0.052-0.385]	0.02 (−0.34 to 0.39)	0.908
CAR-specific AUCi (min·µg/dL)	2.175 [1.302-3.915]	1.860 [0.867-6.214]	0.02 (−0.35 to 0.38)	0.927
CAR-specific AUCg (min·µg/dL)	24.255 [12.792-40.335]	13.598 [8.032-18.924]	0.36 (−0.01 to 0.72)	0.056
Partial waking-to-bed AUCg (min·µg/dL)	665.688 [312.300-1090.115]	317.013 [272.078-437.597]	0.35 (−0.01 to 0.72)	0.059
Relative CAR (%)	27.548 [9.226-75.241]	73.252 [12.877-239.624]	−0.30 (−0.66 to 0.07)	0.110
C1/C2 ratio	1.391 [1.202-2.743]	1.792 [1.531-2.348]	−0.28 (−0.64 to 0.09)	0.135
Serum cortisol (nmol/L)	385.500 [262.100-513.300]	401.400 [312.500-463.500]	−0.01 (−0.38 to 0.35)	0.940
DCS ($\log_2$ µg/dL/hour)	−0.033 [−0.109 to −0.017]	−0.063 [−0.089 to −0.042]	0.29 (−0.08 to 0.65)	0.123

Note: Values are presented as median [IQR]. Comparisons between score-defined active disease and remission were performed using the Mann–Whitney U test in IBD patients who satisfied the sampling-time criteria for time-dependent analyses. IBD = inflammatory bowel disease; UC = ulcerative colitis; CD = Crohn's disease; MES = Mayo endoscopic subscore; SES-CD = Simple Endoscopic Score for Crohn's Disease; CAR = cortisol awakening response; C0 = awakening salivary cortisol; C1 = salivary cortisol at +30-45 min after awakening; C2 = bedtime salivary cortisol; AUCi = area under the curve with respect to increase; AUCg = area under the curve with respect to ground; DCS = diurnal cortisol slope.

https://doi.org/10.3390/medsci14040463

Supplementary Table S3. hs-CRP — Spearman correlations (log_{10}(hs-CRP); IBD time-dependent analytical sample, n = 97)

Cortisol outcome	ρ	Unadj. p	q (panel BH-FDR)	q (global BH-FDR)
C0 (µg/dL)	+0.325	0.001	0.003	0.041
C1 (µg/dL)	+0.185	0.069	0.115	0.672
C2 (µg/dL)	+0.134	0.191	0.239	0.703
ΔCAR (µg/dL)	−0.402	<0.001	0.002	0.002
CAR-specific AUCi (min·µg/dL)	−0.408	<0.001	0.002	0.002
CAR-specific AUCg (min·µg/dL)	+0.238	0.019	0.038	0.377
Partial waking-to-bed AUCg (min·µg/dL)	+0.156	0.126	0.180	0.703
Relative CAR (%)	−0.517	<0.001	0.002	<0.001
C1/C2 ratio	−0.018	0.860	0.945	0.955
Serum cortisol (nmol/L)	+0.007	0.945	0.945	0.991

Note: Associations were assessed using Spearman rank correlation in the IBD time-dependent analytical sample. hs-CRP was log10-transformed for correlation analyses. Unadjusted p-values, within-table Benjamini–Hochberg false discovery rate (BH-FDR) q-values, and global BH-FDR q-values are shown. Global q-values were derived from the prespecified 140-test biomarker-cortisol correlation matrix. hs-CRP = high-sensitivity C-reactive protein; IBD = inflammatory bowel disease; CAR = cortisol awakening response; C0 = awakening salivary cortisol; C1 = salivary cortisol at +30-45 min after awakening; C2 = bedtime salivary cortisol; AUCi = area under the curve with respect to increase; AUCg = area under the curve with respect to ground.

Supplementary Table S4. Faecal calprotectin–Spearman correlations (IBD time-dependent analytical sample, n = 97; µg/g)

Cortisol outcome	ρ	Unadj. p	q (panel BH-FDR)	q (global BH-FDR)
C0 (µg/dL)	+0.268	0.008	0.040	0.187
C1 (µg/dL)	+0.180	0.077	0.110	0.678
C2 (µg/dL)	+0.209	0.040	0.084	0.447
ΔCAR (µg/dL)	−0.183	0.072	0.110	0.672
CAR-specific AUCi (min·µg/dL)	−0.173	0.089	0.111	0.681

https://doi.org/10.3390/medsci14040463

Cortisol outcome	ϱ	Unadj. p	q (panel BH-FDR)	q (global BH-FDR)
CAR-specific AUCg (min·µg/dL)	+0.207	0.042	0.084	0.447
Partial waking-to-bed AUCg (min·µg/dL)	+0.210	0.039	0.084	0.447
Relative CAR (%)	−0.280	0.005	0.040	0.154
C1/C2 ratio	−0.139	0.176	0.196	0.703
Serum cortisol (nmol/L)	+0.002	0.987	0.987	0.994

Note: Associations were assessed using Spearman rank correlation in the IBD time-dependent analytical sample. Unadjusted p-values, within-table Benjamini–Hochberg false discovery rate (BH-FDR) q-values, and global BH-FDR q-values are shown. Global q-values were derived from the prespecified 140-test biomarker–cortisol correlation matrix. IBD = inflammatory bowel disease; CAR = cortisol awakening response; C0 = awakening salivary cortisol; C1 = salivary cortisol at +30-45 min after awakening; C2 = bedtime salivary cortisol; AUCi = area under the curve with respect to increase; AUCg = area under the curve with respect to ground.

Supplementary Table S5. MES (UC only)–Spearman correlations (n = 49)

MES correlations were corrected within 10 UC-specific outcome pairs. These analyses are exploratory and subtype-based; interpretation is limited by the ordinal 0-3 range of the MES.

Cortisol outcome	ϱ	Unadj. p	q (within-table BH-FDR)
C0 (µg/dL)	+0.140	0.344	0.859
C1 (µg/dL)	+0.042	0.777	0.859
C2 (µg/dL)	+0.073	0.621	0.859
ΔCAR (µg/dL)	−0.033	0.825	0.859
CAR-specific AUCi (min·µg/dL)	−0.031	0.835	0.859
CAR-specific AUCg (min·µg/dL)	+0.076	0.608	0.859
Partial waking-to-bed AUCg (min·µg/dL)	+0.072	0.626	0.859
Relative CAR (%)	−0.197	0.180	0.859
C1/C2 ratio	−0.157	0.287	0.859
Serum cortisol (nmol/L)	−0.026	0.859	0.859

Note: Associations were assessed using Spearman rank correlation within the UC subgroup. Unadjusted p-values and within-table Benjamini–Hochberg false discovery rate (BH-FDR) q-values are shown. MES correlations were corrected within the displayed UC-

https://doi.org/10.3390/medsci14040463

specific outcome set. UC = ulcerative colitis; MES = Mayo endoscopic subscore; CAR = cortisol awakening response; C0 = awakening salivary cortisol; C1 = salivary cortisol at +30-45 min after awakening; C2 = bedtime salivary cortisol; AUCi = area under the curve with respect to increase; AUCg = area under the curve with respect to ground.

Supplementary Table S6. SES-CD (CD only)–Spearman correlations (n = 48)

Cortisol outcome	ρ	Unadj. p	q (within-table BH-FDR)
C0 (µg/dL)	−0.001	0.995	0.995
C1 (µg/dL)	−0.114	0.440	0.641
C2 (µg/dL)	+0.097	0.513	0.641
ΔCAR (µg/dL)	−0.178	0.225	0.586
CAR-specific AUCi (min·µg/dL)	−0.186	0.205	0.586
CAR-specific AUCg (min·µg/dL)	−0.106	0.475	0.641
Partial waking-to-bed AUCg (min·µg/dL)	−0.050	0.735	0.817
Relative CAR (%)	−0.161	0.276	0.586
C1/C2 ratio	−0.209	0.155	0.586
Serum cortisol (nmol/L)	−0.155	0.293	0.586

Note: Associations were assessed using Spearman rank correlation within the CD subgroup. Unadjusted p-values and within-table Benjamini–Hochberg false discovery rate (BH-FDR) q-values are shown. SES-CD correlations were corrected within the displayed CD-specific outcome set. CD = Crohn's disease; SES-CD = Simple Endoscopic Score for Crohn's Disease; CAR = cortisol awakening response; C0 = awakening salivary cortisol; C1 = salivary cortisol at +30-45 min after awakening; C2 = bedtime salivary cortisol; AUCi = area under the curve with respect to increase; AUCg = area under the curve with respect to ground.

Supplementary Table S7. Cortisol parameters according to biological therapy status.

Variable	Biological therapy: Yes (n=60)	Biological therapy: No (n=37)	p-value
Serum cortisol (nmol/L)	369 [309-443]	406 [229-565]	0.335
C0 awakening cortisol (µg/dL)	0.365 [0.234-1.07]	0.516 [0.265-1.21]	0.180
C1 +30-45 min cortisol (µg/dL)	0.635 [0.357-1.24]	0.895 [0.491-1.33]	0.243

https://doi.org/10.3390/medsci14040463

Variable	Biological therapy: Yes (n=60)	Biological therapy: No (n=37)	p-value
C2 bedtime cortisol (μg/dL)	0.32 [0.14-0.88]	0.36 [0.113-1.11]	0.978
ΔCAR (μg/dL)	0.14 [0.069-0.289]	0.149 [0.1-0.243]	0.746
CAR-specific AUCi (min·μg/dL)	2.1 [1.18-4.56]	2.43 [1.56-4]	0.628
CAR-specific AUCg (min·μg/dL)	15.7 [10.5-37.7]	22.4 [12.9-40.9]	0.275
Partial waking-to-bed AUCg (min·μg/dL)	440 [267-991]	573 [287-1132]	0.487
Daily amplitude C1−C2 (μg/dL)	0.205 [0.094-0.327]	0.246 [0.162-0.501]	0.044
Relative CAR (%)	32.9 [12.7-120]	26.2 [8.65-82.4]	0.210
C1/C2 ratio	1.41 [1.25-2.75]	1.8 [1.23-5.09]	0.222
DCS (log_2 μg/dL/hour)	-0.038 [-0.107--0.02]	-0.057 [-0.153--0.019]	0.214

Note: Values are presented as median [Q1-Q3]. Comparisons according to current biological therapy status were performed using the Mann–Whitney U test among IBD patients with available treatment data. IBD = inflammatory bowel disease; CAR = cortisol awakening response; C0 = awakening salivary cortisol; C1 = salivary cortisol at +30-45 min after awakening; C2 = bedtime salivary cortisol; AUCi = area under the curve with respect to increase; AUCg = area under the curve with respect to ground; DCS = diurnal cortisol slope.

Supplementary Table S8. Raw-scale HC3 OLS sensitivity models for selected time-dependent cortisol outcomes.

Outcome	Adjusted β	95% CI	p-value	Adjusted R^2
C0 awakening cortisol (μg/dL)	−0.549	−0.782 to −0.316	<0.001	0.107
C1 post-awakening cortisol (μg/dL)	−1.054	−1.307 to −0.801	<0.001	0.295
C2 bedtime cortisol (μg/dL)	−0.009	−0.186 to +0.167	0.917	−0.025
ΔCAR (C1−C0, μg/dL)	−0.505	−0.720 to −0.291	<0.001	0.139
CAR-specific AUCi (min·μg/dL)	−8.139	−11.804 to −4.474	<0.001	0.137
CAR-specific AUCg (min·μg/dL)	−25.808	−33.092 to −18.524	<0.001	0.220

Partial waking-to-bed AUCg (min·μg/dL)	−478.12	−651.96 to −304.28	<0.001	0.151
DCS (log_2 μg/dL/hour)	+0.0818	+0.0518 to +0.1119	<0.001	0.149

Note: Models were adjusted for age, sex, BMI, wake time, and dt1 in the time-dependent analytical sample (n=175). β represents the adjusted raw-scale regression coefficient for IBD status, with healthy controls as the reference group. Negative β values indicate lower adjusted outcome values in IBD patients compared with controls. For DCS, positive β values indicate a less negative, flatter post-awakening-to-bedtime cortisol decline. Confidence intervals and p-values were calculated using HC3 heteroscedasticity-consistent standard errors. Abbreviations: OLS, ordinary least squares; HC3, heteroscedasticity-consistent standard errors; IBD, inflammatory bowel disease; CI, confidence interval; BMI, body mass index; CAR, cortisol awakening response; C0, awakening salivary cortisol; C1, salivary cortisol at +30-45 min after awakening; C2, bedtime salivary cortisol; dt1, C0-to-C1 sampling interval; AUCi, area under the curve with respect to increase; AUCg, area under the curve with respect to ground; DCS, diurnal cortisol slope.

Supplementary Table S9. Median quantile-regression sensitivity models for cortisol outcomes.

	**Adjusted median β**	**95% CI**	**p-value**
C0 awakening cortisol (μg/dL)	−0.597	−0.890 to −0.304	<0.001
C1 post-awakening cortisol (μg/dL)	−1.382	−1.693 to −1.071	<0.001
C2 bedtime cortisol (μg/dL)	+0.018	−0.181 to +0.217	0.860
ΔCAR (C1−C0, μg/dL)	−0.384	−0.477 to −0.291	<0.001
CAR-specific AUCi (min·μg/dL)	−6.478	−7.976 to −4.980	<0.001
CAR-specific AUCg (min·μg/dL)	−29.800	−38.862 to −20.737	<0.001
Partial waking-to-bed AUCg (min·μg/dL)	−589.20	−791.95 to −386.45	<0.001
DCS (log_2 μg/dL/hour)	+0.0965	+0.0652 to +0.1277	<0.001

Note: Median quantile-regression models (τ = 0.50) were adjusted for age, sex, BMI, wake time, and dt1 in the time-dependent analytical sample (n=175). β represents the adjusted median difference for IBD patients compared with healthy controls. Negative β values indicate lower adjusted medians in IBD patients. For DCS, positive β values indicate a less negative, flatter post-awakening-to-bedtime cortisol decline. These models were used as a distribution-robust sensitivity analysis to assess whether the main findings were driven by skewness or extreme values. Abbreviations: IBD, inflammatory bowel disease; CI, confidence interval; BMI, body mass index; CAR, cortisol awakening response; C0, awakening salivary cortisol; C1, salivary cortisol at +30-45 min after awakening; C2, bedtime salivary

cortisol; dt1, C0-to-C1 sampling interval; AUCi, area under the curve with respect to increase; AUCg, area under the curve with respect to ground; DCS, diurnal cortisol slope.

Supplementary Table S10. Repeated-measures mixed model for log-transformed salivary cortisol across C0, C1, and C2

Contrast	Effect estimate	95% CI	p-value
C0: IBD vs. controls	ratio 0.546	0.413-0.723	<0.001
C1: IBD vs. controls	ratio 0.431	0.325-0.570	<0.001
C2: IBD vs. controls	ratio 0.925	0.699-1.225	0.588
IBD × time-point interaction	Wald $\chi^2(2) = 36.34$	/	1.29×10^{-8}

Note: The model included log-transformed salivary cortisol at C0, C1, and C2 as repeated outcomes, a participant-level random intercept, and fixed effects for IBD status, time point, IBD × time-point interaction, age, sex, BMI, wake time, and dt1. The model used the time-dependent analytical sample (n=175 participants; 525 salivary cortisol observations). Time point-specific contrasts are presented as exponentiated IBD/control ratios. Ratios below 1 indicate lower model-estimated cortisol values in IBD patients compared with controls at the corresponding time point. The omnibus IBD × time-point interaction was tested using a Wald χ^2 test. Abbreviations: IBD, inflammatory bowel disease; CI, confidence interval; BMI, body mass index; C0, awakening salivary cortisol; C1, salivary cortisol at +30-45 min after awakening; C2, bedtime salivary cortisol; dt1, C0-to-C1 sampling interval.

Supplementary Table S11. Exploratory comparison of demographic and biochemical characteristics between patients with ulcerative colitis and Crohn's disease.

Parameter	UC (n = 51)	CD (n = 49)	p-value	q-value
Age (years)	38 [29–45.5]	39 [26–49]	0.639	0.852
Sex, male, n (%)	27 (52.9%)	34 (69.4%)	0.139	0.560
Weight (kg)	84.0 ± 10.4	80.1 ± 9.9	0.058	0.560
Height (m)	1.83 [1.76–1.90]	1.79 [1.75–1.87]	0.166	0.560
BMI (kg/m²)	25.08 ± 2.85	24.44 ± 3.17	0.296	0.615
Haemoglobin (g/L)	135 [125.5–149.5]	139 [130–155]	0.196	0.560
Haematocrit (L/L)	0.42 [0.38–0.45]	0.43 [0.40–0.46]	0.242	0.606
Platelets (×10⁹/L)	260 [228.5–305]	254 [223–297]	0.442	0.649
Albumin (g/L)	39 [35.5–42]	41 [39–42]	0.008	0.165
hs-CRP (mg/L)	3.6 [0.7–12.1]	2.5 [0.9–18.0]	0.748	0.890
Iron (µmol/L)	12 [7.25–21]	18 [7–20]	0.369	0.615
Ferritin (µg/L)	29 [10.5–81.5]	35 [15–67]	0.920	0.920
Cholesterol (mmol/L)	4.4 [3.75–5.65]	4.8 [4.0–5.7]	0.454	0.649
LDL (mmol/L)	2.66 [1.95–3.60]	2.80 [2.10–3.60]	0.339	0.615
HDL (mmol/L)	1.30 [1.00–1.60]	1.30 [1.10–1.60]	0.798	0.890
Triglycerides (mmol/L)	1.00 [0.80–1.49]	0.90 [0.80–1.40]	0.895	0.920

https://doi.org/10.3390/medsci14040463

**Parameter**	**UC (n = 51)**	**CD (n = 49)**	**p-value**	**q-value**
ALT (U/L)	17 [12.5–26]	19 [14–27]	0.317	0.615
Urea (mmol/L)	4.4 [3.6–5.25]	4.3 [3.5–5.6]	0.801	0.890
Creatinine (μmol/L)	69 [61–74.5]	72 [65–77]	0.193	0.560
Uric acid (μmol/L)	247 [202–302]	273 [235–303]	0.159	0.560

Note: Data are presented as median [IQR], except weight and BMI, which are presented as mean ± SD, and sex, which is presented as n (%). Unadjusted p-values were obtained using the Mann–Whitney U test for non-normally distributed continuous variables, Welch's independent-samples t-test for weight and BMI, and the chi-square test with continuity correction for sex. q-values were calculated using the Benjamini–Hochberg false-discovery-rate correction across all 20 comparisons. Abbreviations: UC, ulcerative colitis; CD, Crohn's disease; IQR, interquartile range; SD, standard deviation; BMI, body mass index; hs-CRP, high-sensitivity C-reactive protein; LDL, low-density lipoprotein cholesterol; HDL, high-density lipoprotein cholesterol; ALT, alanine aminotransferase; BH-FDR, Benjamini–Hochberg false discovery rate.

Disclaimer/Publisher's Note: The statements, opinions and data contained in all publications are solely those of the individual author(s) and contributor(s) and not of MDPI and/or the editor(s). MDPI and/or the editor(s) disclaim responsibility for any injury to people or property resulting from any ideas, methods, instructions or products referred to in the content.

https://doi.org/10.3390/medsci14040463
